# Epidemiology of periportal fibrosis and relevance of current *Schistosoma mansoni* infection within the context of repeated mass drug administration in rural Uganda: a population-based, cross-sectional study

**DOI:** 10.1016/j.lanmic.2024.07.007

**Published:** 2024-12

**Authors:** Seun Anjorin, Betty Nabatte, Simon Mpooya, Benjamin Tinkitina, Christopher K Opio, Narcis B Kabatereine, Goylette F Chami

**Affiliations:** a Big Data Institute, Nuffield Department of Population Health, University of Oxford, Oxford, UK; b Division of Vector-Borne and Neglected Tropical Diseases Control, Uganda Ministry of Health, Kampala, Uganda; c Aga Khan University Hospital, Nairobi, Kenya

## Abstract

**Background:**

WHO guidelines for schistosomiasis-related morbidity control and elimination rely on current infection as a proxy indicator for morbidity. We evaluated these guidelines within the context of repeated mass drug administration and periportal fibrosis attributable to chronic intestinal schistosomiasis.

**Methods:**

We examined 1442 households randomly sampled from 38 villages in Buliisa, Pakwach, and Mayuge districts of Uganda within the SchistoTrack cohort. Periportal fibrosis was diagnosed in 2834 individuals aged 5–90 years using ultrasound and image patterns C–F from the Niamey protocol. *Schistosoma mansoni* status and intensity were diagnosed by Kato-Katz microscopy and point-of-care circulating cathodic antigen tests. Schistosome infection, co-infections, and comorbidities were examined as exposures for periportal fibrosis. Multivariable logistic regressions were run with SEs clustered by household.

**Findings:**

Between Jan 6 and Feb 3, 2022, 342 (12·1%) of 2834 participants were diagnosed with periportal fibrosis. By Kato-Katz microscopy, 1229 (43·4%) of 2834 participants were infected. 1863 (65·7%) of 2834 participants had trace positive point-of-care circulating cathodic antigen tests, which was higher than prevalence by Kato-Katz microscopy, and 1158 (40·9%) of 2834 participants had trace negative point-of-care circulating cathodic antigen tests. Individual schistosome status, intensity, and prevalence of heavy intensity infections of less than 1% and less than 5% were not correlated with periportal fibrosis likelihood or village prevalence. Periportal fibrosis likelihood linearly increased with age from age 5 years to age 25 years, non-linearly increased from age 26 years to age 45 years, attenuated or remained unchanged from age 46 years to age 60 years, and steadily decreased past 60 years of age. History of liver diseases, HIV, and ultrasound-detected chronic hepatitis or early cirrhosis-like disease were associated with more than two-times increased periportal fibrosis likelihood.

**Interpretation:**

WHO guidelines reliant on current schistosome status and intensity are uninformative for identifying probable cases or communities with periportal fibrosis. History of HIV and underlying chronic hepatitis or early cirrhosis-like disease are risk factors that could be investigated for periportal fibrosis surveillance and management.

**Funding:**

NDPH Pump Priming Fund, Wellcome Trust, John Fell Fund, Robertson Foundation, and UK Research and Innovation Engineering and Physical Sciences Research Council.

## Introduction

52 countries require mass drug administration (MDA) for schistosomiasis, with over 250 million people estimated to be infected and over 700 million people living in endemic areas.^1^ The intestinal forms of chronic schistosomiasis, most prevalent in sub-Saharan Africa where *Schistosoma mansoni* is endemic*,* are characterised by periportal fibrosis.^1^ Mature schistosome flukes live in the mesenteric venules; paired female flukes release approximately 300–400 eggs per day.^2^ Eggs can become lodged in the gut wall or swept back into the vasculature of the liver and spleen. Periportal fibrosis is a chronic hepatic condition caused by an inflammatory response, leading to granulomas and, depending on the host immune response, eventual fibrosis around parasite eggs in the portal segmental branches and eventually the main portal vein. Severe fibrosis can cause blockage of the main portal vein, leading to portal hypertension and portosystemic collaterals, such as oesophageal varices that when ruptured confer a high probability of death.^3^ Despite the clinical severity, there are no routine treatment or case management strategies for individuals with periportal fibrosis. MDA with praziquantel to treat current infections only is the mainstay of treatment for schistosomiasis-associated conditions, with the assumption that early-in-life and frequent praziquantel administration will prevent irreversible sequelae. Because of this assumption, current WHO guidelines for schistosomiasis^4^ set target thresholds for current infection intensity diagnosed via microscopy. Less than 5% and less than 1% prevalence of heavy infection intensity, as measured by 400 or more eggs per g of stool for *S mansoni*, is used as a proxy indicator for the control and elimination of morbidity as a public health problem, respectively.^4^

**Research in context**

**Evidence before this study**

Morbidity due to parasitic infection is a complex interplay of current and past exposures. WHO guidelines for elimination of schistosomiasis as a public health problem assume current infection intensity is a reliable proxy indicator of prevalent morbidity. Community infection thresholds are defined in guidelines and, when met, the assumption is there is no schistosomiasis-related morbidity. There is insufficient evidence for the association of infection with prevalent morbidity in the context of repeated treatment through routine mass drug administration (MDA). To evaluate WHO guidelines, there is a need for large-scale population-based, cross-sectional studies in endemic areas where infection status and intensity are compared with morbidity at the same timepoint. A cross-sectional design enables the investigation of a range of risk factors to assess the relative importance of current schistosome infection. Periportal fibrosis is a schistosomiasis-associated severe morbidity with clinical consequences, such as portal hypertension, upper gastrointestinal tract bleeding, and ultimately premature death. Periportal fibrosis is schistosomiasis-specific or attributable to schistosome pathology, unlike more subtle conditions with complex causes (eg, anaemia). Hence, investigating periportal fibrosis serves as a first-line, conservative approach to evaluating WHO guidelines for elimination of schistosomiasis-related morbidity as a public health problem. A systematic literature search as part of an ongoing meta-analysis was prospectively registered on PROSPERO, CRD42022333919. Databases were searched from inception to May 18, 2022 and included the Cochrane Central Register of Controlled Trials, Embase, Global Health, Global Index Medicus, and MEDLINE. The following general terms were used: “Schistosoma” AND “fibrosis” AND “intensity” AND “infection” AND “periportal OR liver”. Studies were included that considered *Schistosoma mansoni*, *Schistosoma japonicum,* or *Schistosoma mekongi* species. Only original research articles in English were considered. No restriction on date of publication, age, sex, or region was applied. Infection was required to be diagnosed as opposed to self-reported. Periportal fibrosis was defined by the study authors.

Only ten studies on schistosomiasis and periportal fibrosis were initiated (or published) in sub-Saharan Africa from 2003 onwards after the start of MDA. Among those studies, only three analyses were minimally adjusted for age and sex, whereas the remainder of studies presented unadjusted relationships between schistosome infection and periportal fibrosis, ignoring confounders. Studies focused on narrow age groups, leaving open questions as to the age-specific likelihood of periportal fibrosis. Ultrasound data collection protocols and validation across studies were poorly reported. There was a paucity of investigations on co-infections and comorbidities, with only one study adjusting for co-infections. Only one study (Wiegand and colleagues, 2021) evaluated current WHO guidelines for the control and elimination of schistosomiasis-related morbidity as a public health problem. However, administrative MDA data were used with sparse non-random samples focusing on schoolchildren and a limited frequency of the outcome of periportal fibrosis.

**Added value of this study**

We conducted a comprehensive, population-based study of individuals aged 5 years and older who were eligible for MDA in areas with at least 13 rounds of treatment. We found evidence for a lack of association between current *S mansoni* status, infection intensity, and community prevalence with periportal fibrosis likelihood and prevalence when using two routine diagnostics of Kato-Katz microscopy and point-of-care circulating cathodic antigen tests. No support was found for current WHO guidelines, despite our use of arguably the most biologically specific and severe morbidity associated with schistosomiasis. We also characterised the age-specific likelihood of periportal fibrosis, identifying a possible transitional age as young as 25 years. We identified future avenues for research into co-infections such as HIV and hepatitis B, which appeared to affect periportal fibrosis likelihood, even after controlling for a range of biosocial determinants of schistosome infection, treatment, and unrelated liver fibrosis.

**Implications of all the available evidence**

Future work is needed to understand if co-infections alter the pathogenesis of periportal fibrosis. Importantly, current WHO guidelines should be used only for monitoring reduction in schistosome status and infection intensity and should not be used to assess the control or elimination of periportal fibrosis, which was not correlated with current infections.

There is no consensus on the relevance of current schistosome infections for periportal fibrosis.^5^, ^6^, ^7^, ^8^ In the context of MDA and natural fluke death, periportal fibrosis can outlast current schistosome infections.^6^, ^7^, ^8^, ^9^ Existing studies have focused on unrepresentative samples of restricted populations, often targeting only school-aged children.^5^^,^^6^^,^^8^^,^^10^^,^^11^ There are assumptions that the relationship of age with periportal fibrosis is linear to reflect a cumulative history of exposure and chronic infection,^7^^,^^8^^,^^12^ despite intestinal schistosome prevalence or average intensity being non-linearly associated with age. Schistosome infections have been mostly studied in isolation for their associations with clinical liver pathologies,^7^^,^^8^ neglecting co-infections such as malaria or chronic hepatitis B or C and co-occurring conditions such as alcoholism, which might confound or modify periportal fibrosis.

We investigated periportal fibrosis outcomes in a wide cross-section of participants in rural communities within western and eastern Uganda. We aimed to evaluate current WHO guidelines for schistosomiasis-related morbidity within the context of repeated MDA and to establish the epidemiology of periportal fibrosis considering comorbidities and co-infections.

## Methods

### MDA history

This population-based, cross-sectional study formed the baseline survey in the prospective multimorbidity cohort, SchistoTrack,^13^ in Buliisa, Pakwach, and Mayuge districts in Uganda. In these districts, annual school-based and community-based MDA with praziquantel was started in 2003 to target eligible individuals aged at least 5 years, except in 2019, when only school-based MDA was completed. Of the 19 possible annual rounds of MDA before this study, Buliisa and Pakwach districts received only 13 rounds, whereas Mayuge district received 15 rounds. The most recent round of MDA in our study districts was in 2020. The mean administrative treatment coverage for community-based MDA was 79·8% (SD 22·9%) in Buliisa, 80·5% (24·0%) in Pakwach, and 76·2% (12·2%) in Mayuge. Among all rounds of MDA implementation, Pakwach and Buliisa districts missed WHO-suggested targets of 75% treatment coverage by more than 5% for only 1 year, whereas Mayuge district missed this target by more than 5% for 3 non-consecutive years. All study districts had no MDA in 2007 and 2008.

### Participant sampling

Households were sampled using a local register from each of 38 villages. One child aged 5–17 years and one adult aged 18 years or older were selected by the household head or their spouse and invited for clinical assessments. A uniform probability random sampling approach was taken, in which 40 households were sampled from each register, with oversampling of 30 households per village to allow for ineligible households, migrated households no longer in the village, and baseline non-response, unavailability, or refusal. Random numbers were generated using the random package in R version 4.1 out of the total number of households, where the random number corresponded to the order the household was listed in the register. Additional sampling details are provided in the appendix (p 2).

### Ethics approval

Data collection and use were reviewed and approved by Oxford Tropical Research Ethics Committee (OxTREC 509-21), the Vector Control Division Research Ethics Committee of the Uganda Ministry of Health (VCDREC146), and the Uganda National Council of Science and Technology (UNCST HS1664ES). Written informed consent was received from all adults, who also consented on behalf of those younger than 18 years. When possible, children also provided written informed consent. All children provided informed verbal assent.

### Outcome

For periportal fibrosis diagnosis, point-of-care B-mode ultrasound was used to acquire image patterns, following the Niamey protocol and as described in the appendix (p 3).^14^ At least two sonographers were required to jointly scan and agree on grading. All observed patterns of periportal fibrosis were recorded. The highest pattern assigned to each participant was used to construct a binary outcome where patterns C–F were coded as one (periportal fibrosis) and patterns A–B were coded as zero (no periportal fibrosis). A-patterns represented livers with no observed fibrosis. B-patterns were diffuse echogenic small dots or streaks. C-patterns included longitudinal and horizontal cross-sections of segmental branches of the portal tracts showing periportal thickening with no echogenic centres. D-patterns represented extensive echogenic periportal thickening adjacent to the main portal vein. E-patterns and F-patterns included portal fibrosis demonstrating blocked vessels with no observable lumens and extending into the parenchyma and up to the liver edge, respectively. Lumify C5-2 curved linear array transducers (Philips; Amsterdam, Netherlands) were used with the Philips Lumify Ultrasound Application version 3.0 on 8505-F tablets (Lenovo; Beijing, China) with Android Pie version 9.

### Exposures

*S mansoni* infection was diagnosed using Kato-Katz microscopy^15^ with two thick-smear slides from a single stool sample and point-of-care circulating cathodic antigen tests (batch 210811080) from one urine sample taken on the day of clinical examination.^16^ Tr+ and Tr– indicate that trace was or was not included, respectively, as positive for infection status. All schistosome infection indicators are defined in the appendix (p 4).

### Covariates

Detailed covariate definitions are provided in the appendix (p 3). At the individual level, we measured age, self-reported sex where only options of female and male were provided, religion, tribe, water contact, education, and occupation. The history of liver diseases, HIV status, previous use of praziquantel or antiretroviral therapy (ART), complicated or uncomplicated malaria, hepatitis B or C, alcohol use, and smoking were recorded. Ultrasound-based first-pass assessments were completed for ascites, chronic hepatitis or early cirrhosis-like disease, and fatty-like liver, and rapid diagnostic tests of malaria were done (SD Bioline Malaria Ag P.f/Pan; Abbott; Chicago, IL, USA). Patients with chronic hepatitis-like livers were observed to have diffuse, inhomogeneous echogenic nodules and no surface (liver outline) irregularities. Cirrhosis-like livers were defined as having a coarse liver parenchymal echotexture, rounded or blunted caudal liver edges, and potential surface irregularities. At the household level, variables were constructed as described in Chami and colleagues^17^ for total years of settlement in the village, home quality score, electricity availability, social status, home ownership, and improved drinking water, hygiene, and sanitation. Other variables included water body types in the village of residence, availability of at least one public latrine within the village, and district.

### Statistical analysis

The functional form of the relationship between age and periportal fibrosis was identified using locally weighted scatterplot smoothing and generalised additive models, where spans were chosen through 10-fold cross-validation.^18^ To identify key transition points in periportal fibrosis likelihood across age, an alternative method with free-knot spline modelling and generalised cross-validation was used.

Intraclass correlation coefficients in an empty multilevel logistic model were calculated for the clustering of periportal fibrosis within households and villages. Periportal fibrosis predictors were chosen with likelihood ratio tests in univariable models when p≤0·05. To account for the sampling design, fully adjusted multivariable logistic models were run with robust clustered SEs at the household level. Covariates selected by likelihood ratio tests but with a variance inflation factor greater than 10 were removed and presented in alternative models. The model building procedure was rerun for subgroups of only children, adults, and individuals from Pakwach district. Participants with periportal fibrosis, history of liver diseases, chronic hepatitis or early cirrhosis-like disease, or fatty-like livers (Niamey protocol XY patterns) were recoded as zero (no periportal fibrosis) and models were rerun. To assess the predictive capacity of models, 10-fold cross-validation was used with 5-fold cross-validation for subgroup analyses with fewer observations.

All analyses were done in R version 4.1.

### Role of the funding source

The funders of the study had no role in study design, data collection, data analysis, data interpretation, or writing of the report.

## Results

**Figure 1:**
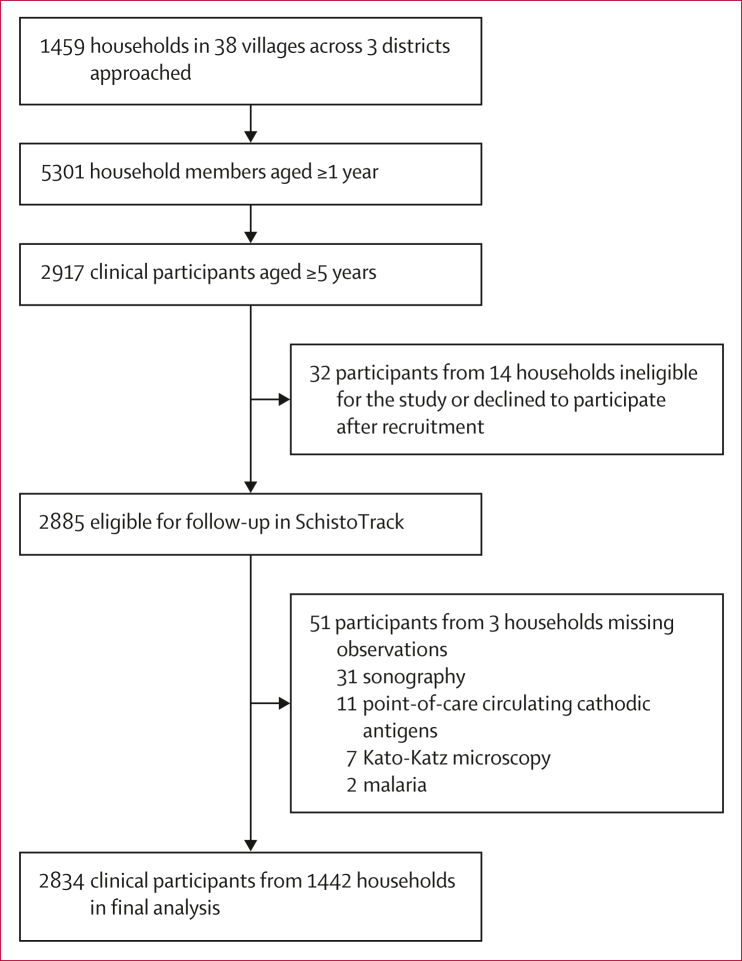
Study flowchart

**Figure 2:**
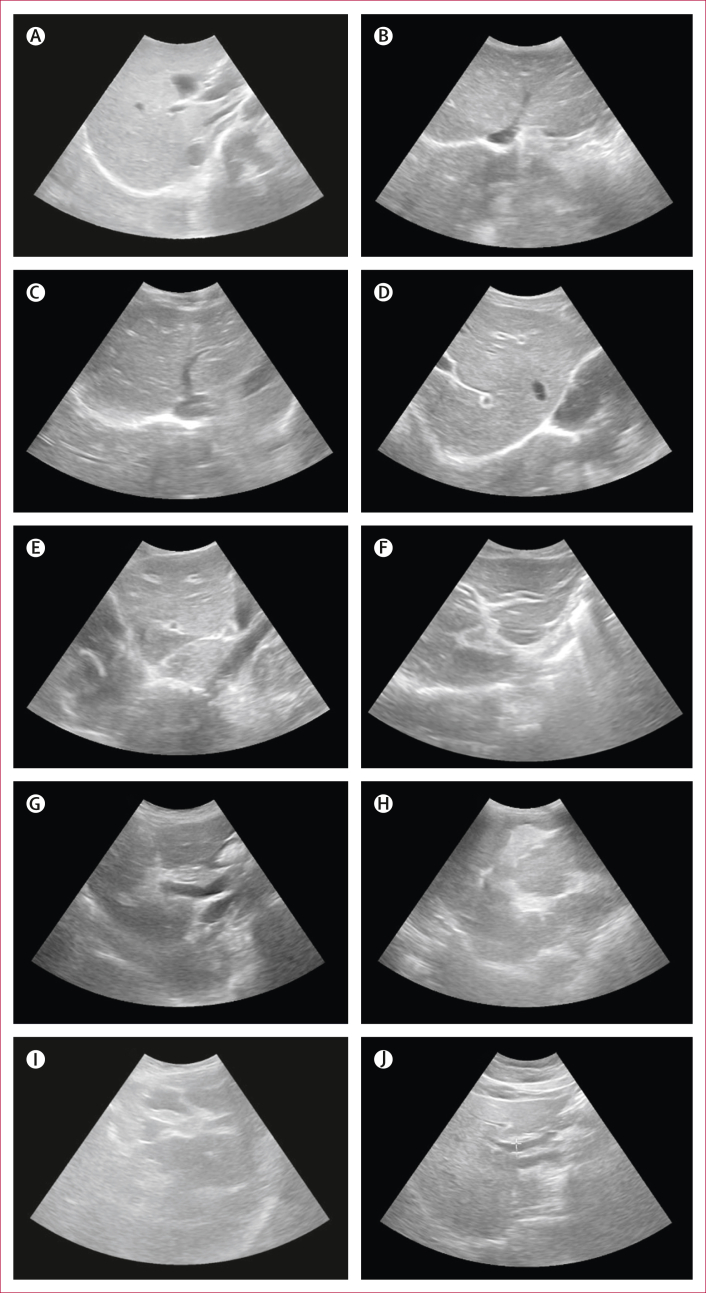
Periportal fibrosis image patterns and comorbidities Patterns as described in the Niamey protocol are shown and suspected diffuse liver diseases such as chronic hepatitis or early cirrhosis-like disease or fatty liver-like disease are noted where observed. (A) Normal liver pattern with no comorbidities. (B) B1 image pattern depicting so-called flying saucers or starry sky. (C) B2 so-called spider thickening pattern. (D) C1 image pattern (prominent peripheral rings) with chronic hepatitis-like liver; the diffuse disease was suspected to be more hepatitis-like in pattern than cirrhosis-like due to the retained liver surface regularity. (E) C1 image pattern with a more cirrhosis-like appearance, possibly advanced cirrhosis, with evidence of a possible shrunken liver and gross surface irregularity. (F) C2 (so-called prominent pipe stems) image pattern. (G) D image pattern (ruff portal bifurcation). (H) Image pattern E (patches representing occluded, bright white vessels). (I) The most severe pattern of periportal fibrosis (F pattern, so-called bird’s claw). (J) Fatty liver-like disease with no evidence of image patterns B–F.

**Figure 3:**
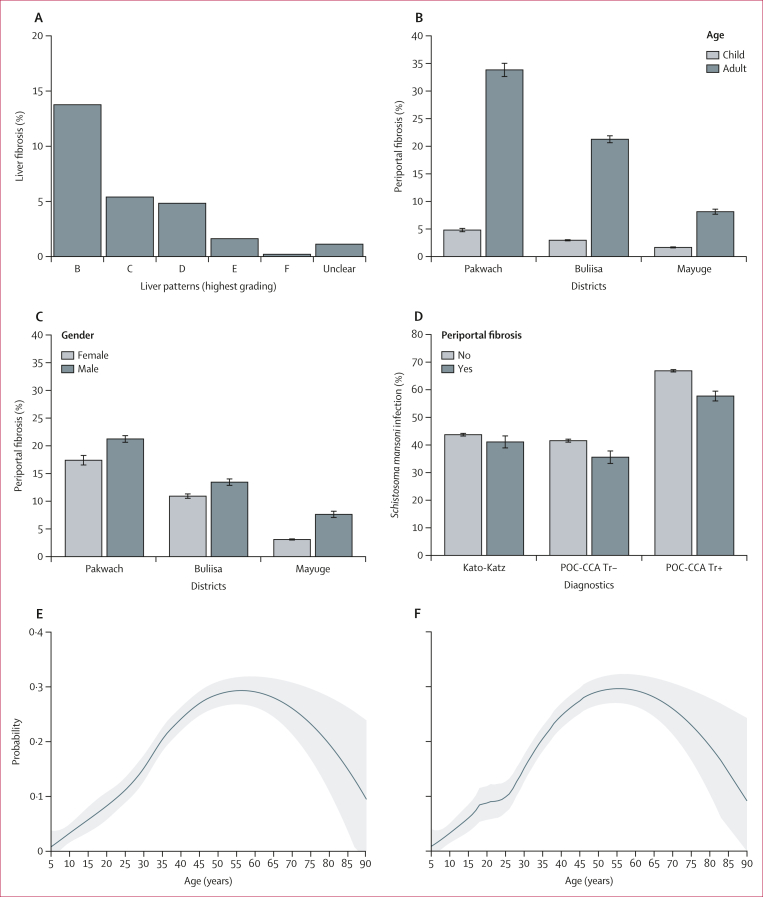
Prevalence of periportal fibrosis and age-specific likelihood Overall N=2834. Pakwach district N=925. Buliisa district N=950. Mayuge district N=959. (A) Prevalence of liver patterns, whereby only the highest pattern per participant was included. (B) Prevalence of periportal fibrosis (liver pattern C–F) by study district and age group. (C) Prevalence of periportal fibrosis by study district and sex. (D) Prevalence of *Schistosoma mansoni* infection by periportal fibrosis. (E) Predicted probability of periportal fibrosis at each age using the locally weighted scatterplot smoothing default span (0·75). (F) Predicted probability of periportal fibrosis at each age using a span of 0·62 selected by 10-fold cross-validation. POC-CCA=point-of-care circulating cathodic antigen test.

Between Jan 6 and Feb 3, 2022, 2834 individuals from 1442 households were clinically examined (figure 1). Participant characteristics are shown in the table with liver fibrosis patterns shown in figure 2. Periportal fibrosis prevalence is shown in figure 3A–D. Household and village covariates are described in the appendix (pp 5–6). 342 (12·1%) of 2834 participants were diagnosed with periportal fibrosis. C image patterns were most common (153 [5·4%] of 2834 participants; figure 3A). The highest prevalence of periportal fibrosis was found in Pakwach district (180 [19·5%] of 925 participants), followed by in Buliisa district (115 [12·1%] of 950 participants), and Mayuge district (47 [4·9%] of 959 participants). Across all individuals, periportal fibrosis prevalence was higher among male participants compared with female participants (figure 2C), and among adults compared with children (figure 3B; table). Five (0·2%) of 2834 participants presented with any grade of ascites; all participants with ascites had periportal fibrosis. 67 (2·3%) of 2834 participants had chronic hepatitis-like disease or early cirrhosis-like disease and 94 (3·3%) of 2834 participants had fatty liver-like disease detected by ultrasound (table). 24 (7·0%) of 342 participants with periportal fibrosis had chronic hepatitis-like disease or early cirrhosis-like disease (table).

**Table:** Participant characteristics

	Overall (n=2834)	No periportal fibrosis (n=2492)	Periportal fibrosis (n=342)
≥1 eggs per g	1229 (43·4%)	1089 (43·7%)	140 (40·9%)
Infection intensity (Kato-Katz microscopy)			
No	1605 (56·6%)	1403 (56·3%)	202 (59·1%)
Low	632 (22·3%)	563 (22·6%)	69 (20·2%)
Mild	364 (12·8%)	320 (12·8%)	44 (12·9%)
High	233 (8·2%)	206 (8·3%)	27 (7·9%)
Eggs per g	0 (0–72)	0 (0–72)	0 (0–60)
Natural log of eggs per g + 1	0 (0–4·29)	0 (0–4·29)	0 (0–4·11)
POC-CCA readings			
Negative	971 (34·3%)	826 (33·1%)	145 (42·4%)
Trace	705 (24·9%)	630 (25·3%)	75 (21·9%)
Pos.1	543 (19·2%)	497 (19·9%)	46 (13·5%)
Pos.2	438 (15·5%)	386 (15·5%)	52 (15·2%)
Pos.3	177 (6·2%)	153 (6·1%)	24 (7·0%)
Infection status (POC-CCA, trace coded as negative)	1158 (40·9%)	1036 (41·6%)	122 (35·7%)
Infection status (POC-CCA, trace coded as positive)	1863 (65·7%)	1666 (66·9%)	197 (57·6%)
History of liver disease	72 (2·5%)	49 (2·0%)	23 (6·7%)
History of HIV	44 (1·6%)	31 (1·2%)	13 (3·8%)
Received antiretroviral therapy	17 (0·6%)	11 (0·4%)	6 (1·8%)
History of hepatitis B or C	25 (0·9%)	21 (0·8%)	4 (1·2%)
PfHRP2	937 (33·1%)	862 (34·6%)	75 (21·9%)
PfHRP2 or pLDH (invalids coded as negative)	1119 (39·5%)	1038 (41·7%)	81 (23·7%)
Malaria (invalids coded as negative)			
Negative	1715 (60·5%)	1454 (58·3%)	261 (76·3%)
PfHRP2 and pLDH, or pLDH only∗	178 (6·3%)	173 (6·9%)	5 (1·5%)
PfHRP2 only	941 (33·2%)	865 (34·7%)	76 (22·2%)
Praziquantel within past year	1341 (47·3%)	1158 (46·5%)	183 (53·5%)
Early cirrhosis-like disease (ultrasound)	5 (0·2%)	3 (0·1%)	2 (0·6%)
Fatty-like liver (ultrasound)	94 (3·3%)	81 (3·3%)	13 (3·8%)
Chronic hepatitis-like disease (ultrasound)	64 (2·3%)	42 (1·7%)	22 (6·4%)
Chronic hepatitis-like disease or early cirrhosis-like disease (ultrasound)	67 (2·4%)	43 (1·7%)	24 (7·0%)
Age, years	18 (9–37)	15 (9–34)	39·5 (30–50)
Adult (aged ≥18 years)	1426 (50·3%)	1128/1426 (79·1%)	298/1426 (20·9%)
Child (aged <18 years)	1408 (49·7%)	1364/1408 (96·9%)	44/1408 (3·1%)
Sex			
Male	1277 (45·1%)	1089/1277 (85·3%)	188/1277 (14·7%)
Female	1557 (54·9%)	1403/1557 (90·1%)	154/1557 (9·9%)
Female adult (aged ≥18 years)	895/1426 (62·8%)	762/895 (85·1%)	133/895 (14·9%)
Male adult (aged ≥18 years)	531/1426 (37·2%)	366/531 (68·9%)	165/531 (31·1%)
Female child (aged <18 years)	662/1408 (47·0%)	641/662 (96·8%)	21/662 (3·2%)
Male child (aged <18 years)	746/1408 (53·0%)	723/746 (96·9%)	23/746 (3·1%)
Religion			
Christian	2361 (83·3%)	2054 (82·4%)	307 (89·8%)
Muslim	456 (16·1%)	425 (17·1%)	31 (9·1%)
Other religion or no religion	17 (0·6%)	13 (0·5%)	4 (1·2%)
Majority religion	2057 (72·6%)	1789 (71·8%)	268 (78·4%)
Majority tribe	2080 (73·4%)	1797 (72·1%)	283 (82·7%)
Tribes			
Other	601 (21·2%)	558 (22·4%)	43 (12·6%)
Musoga	462 (16·3%)	443 (17·8%)	19 (5·6%)
Bagungu	237 (8·4%)	207 (8·3%)	30 (8·8%)
Alur	1534 (54·1%)	1284 (51·5%)	250 (73·1%)
Years of education attained	3 (1–5)	3 (1–5)	4 (2–6)
Occupation			
Other occupation or no occupation	1978 (69·8%)	1819 (73·0%)	159 (46·5%)
Subsistence farmer	500 (17·6%)	430 (17·3%)	70 (20·5%)
Fisherman	248 (8·8%)	154 (6·2%)	94 (27·5%)
Fishmonger	108 (3·8%)	89 (3·6%)	19 (5·6%)
Current drinker	235/2106 (11·2%)	165/1777 (9·3%)	70/329 (21·3%)
Current smoker	157/2106 (7·5%)	110/1777 (6·2%)	47/329 (14·3%)
Any current water contact	1327 (46·8%)	1109 (44·5%)	218 (63·7%)

Data are n (%), median (IQR), or n/N (%). PfHRP2=*Plasmodium falciparum* antigen histidine rich protein 2. pLDH=parasite lactase dehydrogenase. POC-CCA=point-of-care circulating cathodic antigen test.

∗ For pLDH only without PfHRP2, there were only four cases.

By Kato-Katz microscopy, 1229 (43·4%) of 2834 participants were infected, with 233 (8·2%) participants heavily infected (≥400 eggs per g; table). 1863 (65·7%) of 2834 participants had positive point-of-care circulating cathodic antigen tests, which was higher than prevalence by Kato-Katz microscopy, and 1158 (40·9%) of 2834 participants had negative point-of-care circulating cathodic antigen tests, which was similar to prevalence by Kato-Katz microscopy. By district, prevalence by Kato-Katz microscopy was highest in Pakwach (470 [50·8%] of 925 participants) compared with 419 (44·1%) of 950 participants in Buliisa and 340 (35·5%) of 959 participants in Mayuge; the magnitude of the differences in prevalence was similar to negative point-of-care circulating cathodic antigen tests. However, districts were similar with overall prevalence by positive point-of-care circulating cathodic antigen tests with 645 (67·9%) of 650 participants in Buliisa, 629 (68·0%) of 925 participants in Pakwach, and 589 (61·4%) of 959 participants in Mayuge.

Study participants had a median age of 18 years (IQR 9–37), with a range of 5–90 years. Age showed a linear relationship from childhood through to young adulthood (5–25 years), with the likelihood of periportal fibrosis increasing non-linearly from young adulthood to middle age (26–45 years), after which the likelihood of periportal fibrosis becomes steady until around 55–60 years of age, where there is a sharp decline in the likelihood of periportal fibrosis approaching less than 10% (figure 3E, F). A transitional point in periportal fibrosis likelihood was observed around age 25–26 years. Both locally weighted scatterplot smoothing and generalised additive model analyses suggested non-monotonic relationships with an approximately quadratic relationship (inverse U) of age with periportal fibrosis (appendix p 7).

**Figure 4:**
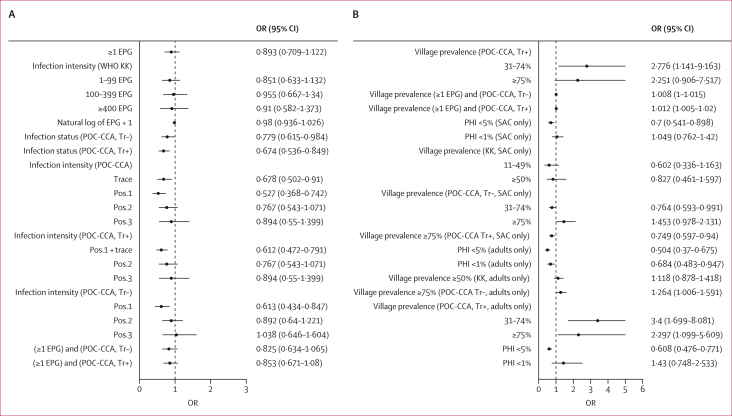
Unadjusted models for *Schistosoma mansoni* infections and periportal fibrosis ORs and corresponding 95% CIs represent univariable logistic regression models. (A) Unadjusted relationships between individual infection indicators and periportal fibrosis. (B) Unadjusted relationships between village-level infection indicators and periportal fibrosis. EPG=eggs per g. KK=Kato-Katz microscopy. OR=odds ratio. PHI=prevalence of heavy infection. POC-CCA=point-of-care circulating cathodic antigen test. SAC=school-aged children.

**Figure 5:**
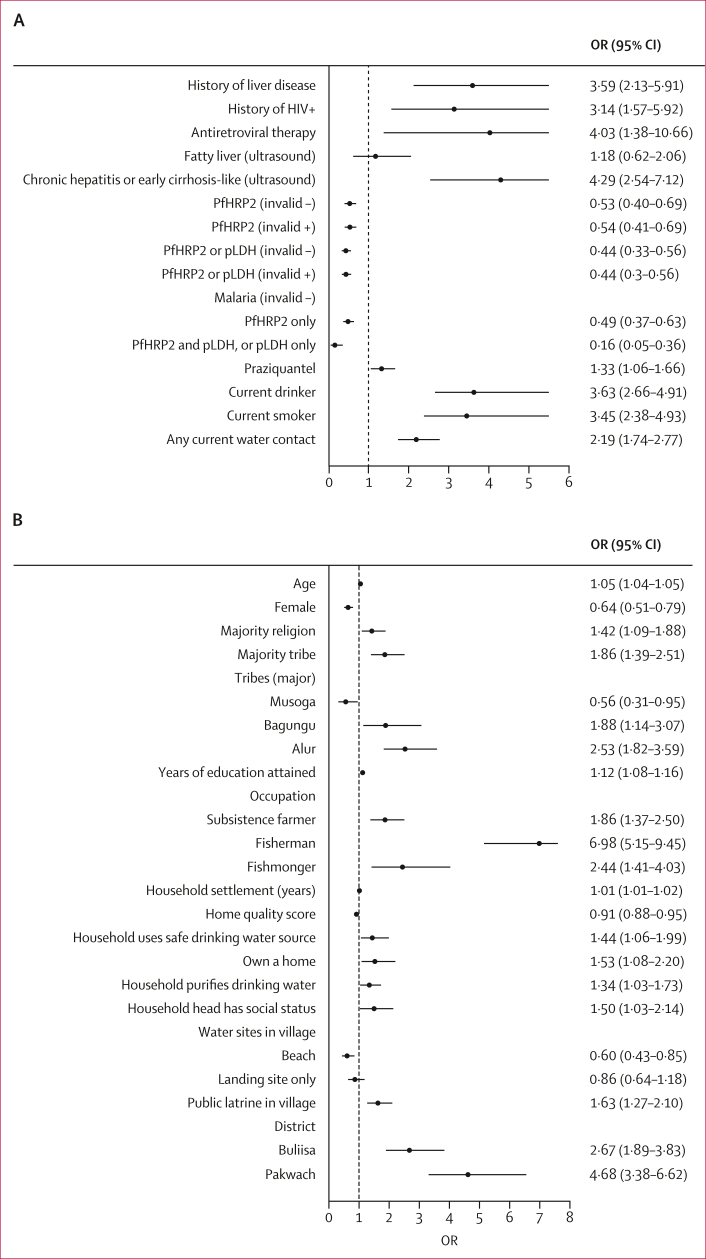
Unadjusted models for covariates and periportal fibrosis ORs and corresponding 95% CIs represent univariable logistic regression models. (A) Unadjusted relationships between periportal fibrosis and co-infections and other medical history factors. Alcohol and smoking variables were run for adult only models (individuals aged ≥18 years). (B) Unadjusted relationships between periportal fibrosis and sociodemographic and ecological indicators. OR=odds ratio. PfHRP2=*Plasmodium falciparum* antigen histidine rich protein 2. pLDH=parasite lactase dehydrogenase.

**Figure 6:**
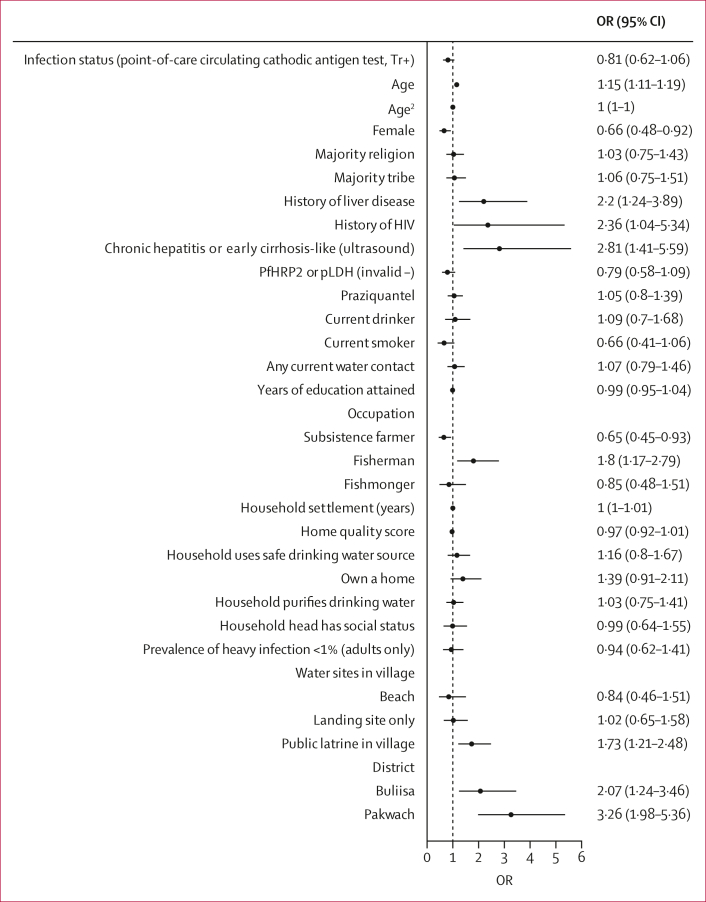
Determinants of periportal fibrosis Fully adjusted model with variables selected through likelihood ratio tests with p≤0·05. No variables in the model had a variance inflation factor greater than ten. OR=odds ratio. PfHRP2=*Plasmodium falciparum* antigen histidine rich protein 2. pLDH=parasite lactase dehydrogenase (generic to all malaria species).

No intraclass clustering of periportal fibrosis was observed within households, whereas 14% of variation was found within villages (figures 4, 5). In fully adjusted models (figure 6; alternative indicators shown in appendix pp 8–9), no individual-level *S mansoni* infection indicators were associated with periportal fibrosis; even in unadjusted models, eggs per g was uncorrelated with periportal fibrosis. This finding was robust to subgroup analyses for children, adults, Pakwach district, and recoding of comorbid diffuse liver disease (XY patterns) as no periportal fibrosis (appendix pp 10–13). Each 1-year increase in age was associated with an increase in the likelihood of periportal fibrosis (odds ratio [OR] 1·15, 95% CI 1·11–1·19) from 5–45 years of age (figure 6). Beyond this age range, the quadratic term for age showed a negative association with periportal fibrosis, although this effect was very small. Female participants were less likely than males to have periportal fibrosis (OR 0·66, 95% CI 0·48–0·92). Fishermen were more likely than unemployed or other occupations to have periportal fibrosis (1·80, 1·17–2·79). Most fishermen were male (244 [98·4%] of 248 participants); most fishmongers were female (91 [84·3%] of 108 participants). Subsistence farmers were less likely to have periportal fibrosis when compared with unemployed individuals or those with other occupations (OR 0·65, 95% CI 0·45–0·93). Participants with a previous diagnosis of liver disease (2·20, 1·24–3·89) and individuals who were HIV positive (2·36, 1·04–5·34) had an increased likelihood of periportal fibrosis. Current ART uptake was not associated with periportal fibrosis (appendix p 9). Participants with ultrasound-detected chronic hepatitis or early cirrhosis-like disease had a higher likelihood of periportal fibrosis (2·81, 1·41–5·34) than individuals without ultrasound-detected diffuse liver diseases. Malaria infection, current drinking, and receipt of praziquantel were not associated with periportal fibrosis. Current drinking was not significant in adjusted models without controlling for chronic hepatitis or early cirrhosis-like disease. No household-level factors were significantly associated with periportal fibrosis.

Village and district characteristics apart from prevalence of heavy infection were associated with periportal fibrosis. Prevalence of heavy infection of less than 1% in children, adults, or all participants was not associated with periportal fibrosis (figure 6; appendix p 8), although prevalence of heavy infection of less than 5% in adults only had a significant association with periportal fibrosis (appendix p 8). Prevalence of heavy infection of less than 1% and of less than 5% were not associated with set levels of periportal fibrosis village prevalence (appendix pp 14–15) and prevalence of heavy infection in school-aged children was only moderately correlated with prevalence of heavy infection in adults by village (appendix pp 16–17). Living in a village with at least one public latrine compared to a village with no public latrines was associated with a higher likelihood of individual periportal fibrosis (OR 1·73, 95% CI 1·21–2·48; figure 6). Participants living in Buliisa and Pakwach had higher likelihood of periportal fibrosis than participants living in Mayuge district (figure 6). When exact tribe was investigated (appendix p 9), participants from the Alur tribe were found to be more likely to have periportal fibrosis compared with other tribes. Alur and Bagungu tribes were similar with respect to the percentage of fishermen (167 [10·9%] of 1534 participants *vs* 26 [11·0%] of 237 participants), whereas the Musoga tribe had fewer fishermen (20 [4·3%] of 462 participants). The tribes of Alur, Bagungu, and Musoga were highly collinear with district effects (variance inflation factor >10). All models showed high predictive accuracy of periportal fibrosis. The mean area under the receiver operating characteristic curve (by ten-fold cross-validation) was 0·82. The Hausman test result of an empty random versus village fixed-effects model (with 38 villages) was χ^2^=3·90, (p<0·05).

## Discussion

Current WHO guidelines prioritise monitoring schistosome infection for assessing progress towards morbidity control and elimination.^4^ In this population-based study, we found little evidence for infection as a proxy indicator for severe morbidity related to intestinal schistosomiasis. Underlying liver diseases, as opposed to current *S mansoni* infections, were key predictors of periportal fibrosis.

Age-specific periportal fibrosis likelihood was non-linear, suggesting the association between age and periportal fibrosis changes over the life course of an individual. The trends we found for periportal fibrosis likelihood do not support well established age-specific infection curves.^19^ Age-specific infection prevalence and mean intensity are known to peak in school-aged children and decline sharply in adults, which has provided previous evidence for acquired immunity, even in the context of steady infection exposure.^20^ However, periportal fibrosis likelihood appears to linearly increase with age in children, whereas the increase is non-linear in young adults. We observed a proximal transitional age around 25–26 years for periportal fibrosis likelihood, which levelled off in older adults and sharply declined in the oldest individuals. Notably, individuals aged 25–26 years would have been aged 9–12 years during 2007–08 when there was no MDA within our study districts, corresponding to missing treatment during the years of life when mean infection intensity was highest. These results suggest that the timing of treatment rather than the duration or frequency of MDA might affect periportal fibrosis development. Other factors unrelated to the cumulative history of schistosome infection might co-occur during late adolescence and affect periportal fibrosis in early adulthood, such as alcohol use or exposures to sexually transmitted pathogens including HIV and hepatitis B.^21^ The drastic decline in periportal fibrosis in older age, beginning from age 55–60 years, might indicate excess mortality due to *S mansoni-*associated morbidities in our study areas, as the median life expectancy in rural Uganda is 65 years.^22^ Future work is ongoing in SchistoTrack to identify the risk factors that individuals encounter over their life course, the onset of periportal fibrosis, and the likelihood of death due to periportal fibrosis.

Prevalence of heavy infection less than 1% and prevalence of heavy infection less than 5% in either school-aged children or adults were poor proxy indicators of the likelihood of an individual—whether another child or adult—having periportal fibrosis and were not associated with the elimination (nor even indicative of any threshold) of periportal fibrosis village prevalence. Village infection prevalence (of any infection intensity), including groupings of WHO categories used to guide the frequency of MDA, was not consistently associated with individual periportal fibrosis likelihood.^4^ WHO guidelines^4^ that use prevalence of heavy infection as a proxy indicator for morbidity burden were uninformative for periportal fibrosis and our results indicate that current guidance^4^ for implementing MDA cannot be used for hotspot identification of communities with a probable high burden of periportal fibrosis.

The long history of MDA (≥13 annual rounds over 19 years) in our study areas coupled with high administrative coverage (>75%) and poor cure rates with praziquantel further suggest that blanket treatment campaigns with praziquantel cannot geographically eliminate periportal fibrosis and there might be limitations of MDA for controlling infection prevalence in high-transmission settings.^23^ Despite MDA, infection prevalence by Kato-Katz microscopy remained high across eastern and western Uganda. At an individual level, there was no association of current status and intensity with periportal fibrosis, irrespective of infection diagnostic. This absence of association was robust even for models with only adults. With no MDA for over a year before the study, the infections diagnosed through routine methods were assumed to be active infections with living adult flukes producing gut antigens and eggs. Hence, our results suggest that targeting active infections through MDA might not target treatment to individuals most likely to have periportal fibrosis. Longitudinal studies are needed to evaluate the effect of repeated MDA on infection and periportal fibrosis prevalence. A limitation of our cross-sectional design was that we were unable to identify whether current infections represent chronic infections. Due to the long lifespan of flukes, current infection status might approximate long-term active infections, residual infection after historical praziquantel treatment, or reinfection. A prospective design is needed to identify the directionality of periportal fibrosis with chronic hepatitis or early cirrhosis-like disease.

Periportal fibrosis shares some epidemiology with current *S mansoni* infection with respect to risk factors, yet has little overlap with predictors of praziquantel receipt. Age, sex, and being a fisherman were significant predictors of periportal fibrosis and in the same directions as expected for predicting infection likelihood or exposure.^19^ However, the effect sizes were approximately a third or less the size of the effects for HIV history, chronic-hepatitis or early cirrhosis-like disease, and study district. Being a fisherman involves water contact and, therefore, infection or reinfection risk.^19^ In our study, the influence of sex was dependent on being a fisherman. Key praziquantel treatment predictors including social status, home quality (wealth), education, and home ownership, among other factors, were not relevant for predicting periportal fibrosis.^17^ This finding might suggest that both direct and indirect measurements of past praziquantel treatment show no correlation with periportal fibrosis, especially severe periportal fibrosis.^24^

Commonly assessed co-occurring conditions, such as alcohol use and malaria, were not associated with periportal fibrosis, despite correlations with periportal fibrosis in other studies that did not adjust for diffuse liver diseases or HIV history.^25^^,^^26^ The correlation of alcoholism with periportal fibrosis might have a lagged effect, where drinking in adolescence instead of current alcohol use might affect periportal fibrosis likelihood in early adulthood.^21^ The absence of association between current drinking and periportal fibrosis was not due to accounting for a mediator of chronic hepatitis or early cirrhosis-like disease; when this variable was removed from the model, current drinking remained non-significant. For malaria non-significance for periportal fibrosis, the parasite predominantly affects the spleen. The liver is only briefly involved in the lifecycle, where sporozoites mature into schizonts, especially for *P falciparum*, which was most common in our study areas. The peak of malaria prevalence and its associated severe morbidities often are amenable to antimalarials and found in children younger than 5 years, who were not studied here.

History of HIV and ultrasound-detected chronic hepatitis or early cirrhosis-like disease were positively correlated with periportal fibrosis. HIV has been shown to correlate with hyaluronic acid concentrations,^27^ which are indicative of liver fibrosis. However, in severe disease stages of HIV, CD4 cells are depleted, which would reduce the ability of the host to mount a granulomatous response to eggs. In the context of widespread ART availability and individuals living longer with HIV, it remains unclear whether HIV or specific ART regimens (and hepatotoxicity) were associated with periportal fibrosis likelihood. Current ART uptake alone was not associated with periportal fibrosis. For HIV, our effects might be underestimated if status was under-reported for individuals who had yet to be diagnosed or who had concerns in reporting due to community stigmatisation. Confirmatory testing that considers viral hepatitis diagnostics, alternative imaging modalities, and fibrosis scores is needed for distinguishing and staging hepatitis from cirrhosis. Considering causes of hepatitis-like livers, hepatitis C is rare in our study areas, whereas hepatitis B, which is estimated to affect more than 10% of adults in western Uganda, could be a promising line of investigation.^28^ For future investigations of periportal fibrosis interactions with diffuse liver diseases, an important consideration is to separate image pattern outcomes. Host immunity is also an important mediating factor for the effects of past exposure and co-infections on periportal fibrosis development and requires more research.^29^

We found that individuals in Pakwach and Buliisa districts were more likely to have periportal fibrosis than individuals in Mayuge district. Geographical differences in periportal fibrosis have been documented previously between western and eastern Uganda.^12^ Our results suggest the district effects are not due to environmental conditions and might be caused by differences in parasite strains. No ecological predictors were correlated with periportal fibrosis. Moreover, individual tribes were highly collinear with district effects. Although there was no household clustering of periportal fibrosis, we are unable to make any inferences on the relevance of genetic ties for periportal fibrosis as we did not require the sampled adult to be the parent of the sampled child. To identify any shared genetic effects through tribe, there is a need to prospectively examine only child–parent pairs within later years of SchistoTrack. Alternatively, the effect of tribe might represent cultural differences in water contact behaviours not captured by differences in the prevalence of fishermen.^30^

In conclusion, monitoring of current schistosome status and infection intensity underpins current WHO guidelines for approximating schistosomiasis-related morbidity, but was uninformative for identifying individuals or communities with periportal fibrosis within the context of repeated MDA. Programmes aimed at controlling periportal fibrosis should explore an integrated case management approach, especially for adults in endemic settings. Within the context of repeated MDA with praziquantel, periportal fibrosis persists. Improved access to health-care services for the treatment of schistosomiasis and capacity building within local health systems for diagnosing and managing periportal fibrosis are needed.

## Declaration of interests

We declare no competing interests.
