# Supplementary material for: Epidemiology of periportal fibrosis and relevance of current Schistosoma mansoni infection within the context of repeated mass drug administration in rural Uganda: a population-based, cross-sectional study
Source: Lancet Microbe. 2024 Dec;5(12):None. doi: 10.1016/j.lanmic.2024.07.007 (PMC11913736; doi:10.1016/j.lanmic.2024.07.007)
Supplement: Supplementary appendix [file mmc1.pdf]

THE LANCET
Microbe

Supplementary appendix

This appendix formed part of the original submission and has been peer reviewed.
We post it as supplied by the authors.

Supplement to: Anjorin S, Nabatte B, Mpooya S, et al. Epidemiology of periportal fibrosis and relevance of current *Schistosoma mansoni* infection within the context of repeated mass drug administration in rural Uganda: a population-based, cross-sectional study. *Lancet Microbe* 2024. https://doi.org/10.1016/j.lanmic.2024.07.007

Appendix for "The epidemiology of periportal fibrosis and relevance of current *Schistosoma mansoni* infection within the context of repeated mass drug administration: a population-based, cross-sectional study in rural Uganda"

Text S1 Supplementary methods

Sampling

Villages within this study were a census of shoreline communities that were eligible for MDA within district sub-counties that had a previously documented high schistosome infection prevalence (>50%). Selected villages within each district were adjacent to one another. Households were first enumerated using household registers kept by the local council secretary of the village (lowest level of government). When there was no village register, neglected tropical disease registers were used that had been provided to village health team members by the Uganda Ministry of Health to enumerate households for MDA—last having been done in early 2020 in the study districts. A uniform probability random sampling approach was taken where 40 households were sampled from each register with an oversampling of 30 households per village to allow for ineligible households, migrated households no longer in the village, and baseline nonresponse/unavailability/refusal. Random numbers were generated out of the total number of households where the random number corresponded to the order the household was listed in the register. As these registers were not necessarily up-to-date, village health team members went door-to-door to enumerate the total number of households in each village and to identify any migrated households from the village registers.

Our lead surveyor (BT) visited the villages to sit with village health team members and village leaders (including the local council 1 chairmen) to identify whether the households met the study eligibility criteria and were still residing in the village. The eligibility criteria included: 1) having a child aged 5-17 years in the home as well as an adult aged 18+ years, 2) having been settled in the village for at least six months and remaining in the village for at least six months of the year, and 3) having not already been sampled from the same family unit compound (i.e. here it was where one male head of household had multiple wives living in the same village in different physical homes). Despite an eligibility requirement to have an adult and child in the home, due to nonresponse such as children noted as available yet sent away to boarding school or due to missing data, it was possible to have households with only one participant selected for clinically assessments. In the case of villages not having at least 40 eligible consenting households, smaller adjacent villages were combined, and 20 households were targeted from each. These villages often had less than 70 households in total. Within each household, one child (5-17 years) and one adult (18+ years) were enrolled in the clinical surveys where the head of household and usually lead wife selected both individuals. Information also was collected on all individuals who were not selected for clinical assessments but were aged 1+ years within the sampled households. The individual eligibility criteria included age, being able to perform daily functions (i.e., not severely ill or hospitalised at the time of visit), and not being drunk (to safeguard the study team). Information was collected on all other household members aged 1+ years. Other than age, which was part of the selection criteria, there was no evidence of sociodemographic differences in people selected versus not selected within the household.

The number of individuals (80/sampling unit) was calculated to detect a minimum effect size of ~8% in an unevenly schistosome exposed/unexposed population (0·57) with a household design effect (1·136) and unbalanced strata/clusters at 97·5 power to account for multiple comparisons.

Variable definitions

All surveys were completed electronically using Open Data Kit v2022.3.6 with Open Data Kit Briefcase v1.17.4. Sociodemographic characteristics, socioeconomic information, medical history, and previous treatment with praziquantel or anti-retroviral therapies were recorded using locally validated household surveys in either Alur, Lugungu, or Lusoga and administered by trained surveyors local to the districts in a language understood by study participants. Interviews were conducted in private within the homes of participants; adults provided information on behalf of children. Age was a continuous variable to the nearest year. Age squared was incorporated in models to account for nonlinearity with PPF. Gender and religion were defined as binary variables with females and Muslims coded as one, respectively. The majority religion was a binary variable, which was coded as one if participants belonged to the majority religion within their own village. Education was defined as a count variable from 1-14, whereas 1-7 represented primary school years, 8-13 captured lower and upper secondary school and 14 represented participants that have completed a post-secondary education. Education also was expressed as a categorical variable of no education, primary education, and secondary education or higher. Occupation was defined as a categorical variable of subsistence farmer, fishermen, fishmongers and no and other occupations as reference category.

Medical history was recorded by household surveyors who used modified versions of the World Health Surveys to collect information on past diagnoses.[1] Individuals were asked whether a government health worker had diagnosed them with a set of conditions. Each condition was constructed as a binary variable. The conditions

asked about included liver diseases (cirrhosis, scarring or fibrosis), HIV, and hepatitis B/C. The WHO Stepwise approach for risk factor surveillance (STEPS) was used to gather information on non-communicable diseases such as smoking and alcohol drinkers.[2] Individuals were coded as current smokers if any tobacco products were used within the past 12 months preceding the study. Individuals were coded as current drinkers if they had consumed any alcohol within the past 12 months. Only study participants aged 10+ years were surveyed on alcohol and smoking behaviours; therefore, these variables were only used in the adult sub-group model.

Previous treatment with praziquantel was coded as a binary variable where participants who received or ingested praziquantel in the past one year were coded as one. Note, there was no MDA (both school and community-based) since early 2020 before our study due to COVID-19 lockdowns, so receipt of praziquantel would have been through other means such as leftover medicines remaining with community medicine distributors or re-allocated to primary health care facilities. The administrative treatment coverage reported in the main text Methods was gathered from records (unpublished) held within the study districts and at the Uganda Ministry of Heatlh, specifically the Division of Vector Borne Diseases and Neglected Tropical Diseases which was responsible for MDA implementation. Current anti-retroviral therapy was also coded as one if participants confirmed they have taken western medicine in the past one month and anti-retroviral therapy was indicated as one of them.

Sonographers regardless of experience with the Niamey Protocol were retrained as part of the study to assess liver patterns as described in the Niamey Protocol. Inter-observer agreement among the eight study sonographers was evaluated to reach a consensus on abdominal sweep procedures for acquiring each PPF pattern. Lossless DICOM images of liver patterns and videos from liver sweeps were saved for quality assurance. Information also was gathered on suspected current diffuse liver diseases. Chronic hepatitis-like, cirrhosis-like were characterised as defined in the main text Methods.[3] Possible shrunken livers or reduced left and right medial sections were considered albeit not required for our definitions of cirrhosis-like livers as we did not only record advanced cases. As chronic hepatitis can mimic early/compensated cirrhosis when diagnosed only via ultrasound,[4] a binary variable equal to one was generated if there was any evidence of either chronic hepatitis-like or cirrhosis-like livers. In our data, all five cases of cirrhosis-like livers had sharp caudal edges suggesting early cirrhosis. Only one of five cases of cirrhosis-like livers had any (here, slightly serrated) surface irregularities. As we were interested in chronic underlying liver diseases, separate 'starry-like' patterns for acute hepatitis were not investigated outside the image pattern B provided in the Niamey Protocol. Fatty-like livers were defined as having diffuse parenchymal brightness with observed liver-to-kidney contrast and posterior attenuation. B-mode ultrasound has shown to be reliable for steatosis exceeding 20%,[5] hence we were likely to miss mild fatty-like liver cases. Ascites was noted when fluid build-up was observed in any of the four abdominal quadrants. If fluid was observed, but not present in all four quadrants then it was graded as mild. If fluid was observed in all four quadrants, but the abdomen and organs were still clearly distinguishable then ascites was graded as moderate. When there was fluid build-up in all four quadrants to the point that the quadrants were indistinguishable, and organs were difficult to differentiate then ascites was graded as severe.

Using also the household survey, water contact was recorded and household-level variables were observed. Any water contact was coded as one if a participant did at least one of following activities on a weekly basis in the lake or river near their village including fishing, fishmongering, collecting papyrus/shells, retrieving drinking water, washing clothes with/without soap, washing jerry cans or other household supplies, swimming, or playing in water. Household-level variables including water, sanitation, and hygiene indicators, social status, home quality, electricity, ownership, and number of rooms as well as village ecology and infrastructure were defined as described in Chami et al. 2016.[6] In brief, social status was coded at the household level as a binary variable and was positive if any adult in the home previously had or currently held at the time of survey a position of status within their village that included membership to the local council (local government), beach management committee, village health team, or religious, clan, or tribe leadership. Improved water, sanitation, and hygiene were defined as indicated in the World Health Organization and United Nations Children's Fund Joint Monitoring Programme Standards for improved access.[7] The standards are based on sufficient water quantity, safety of source of water, privacy and type of latrine, as well as the availability of soap within homes. The years the household had settled in the village was a continuous variable defined as the years anyone in the household or an immediate relative of the household head (who was in the same household) had been living in the village. Two outliers/typos in this variable were recoded to the next nearest value. The recoded outliers were -12 and 3333501, which were recoded to 12 and 50, respectively. Village-level variables such as the infrastructure available (e.g. public tap or public latrine) were recorded by enumerators who physically moved around each village to inspect the resources and take waypoints of their locations.

Table S1 *S. mansoni* infection indicators.

Infection Indicator	Definition
LN(EPG + 1)	Natural log of average egg per gram of stool (+1)
Infection intensity (POC-CCA, Tr+)	Infection categories from urine sample using point-of-care circulating cathodic antigen (POC-CCA) test; trace positive
Infection intensity (POC-CCA, Tr-)	Infection categories from urine sample using POC-CCA test; trace negative
Village prev. (KK)	Village level prevalence of infection by KK
Village prev. ≥50% (KK)	Village prevalence setting of schistosomiasis infection by KK.
Village prev. (POC-CCA, Tr-)	Village level prevalence of infection by POC-CCA trace negative.
Village prev. (POC-CCA, Tr+)	Village level prevalence of infection by POC-CCA trace positive; continuous
Village prev. ≥31% (POC-CCA, Tr-)	Village prevalence setting of schistosomiasis infection by POC-CCA trace negative.
Village prev. (POC-CCA, Tr+)	Village prevalence for *S. mansoni* infection by POC-CCA trace positive.
Village prev. (≥ 1 EPG) & (POC-CCA, Tr-)	Village-level infection prevalence by agreement of KK and POC-CCA trace negative
Village prev. (≥ 1 EPG) & (POC-CCA, Tr+)	Village-level infection prevalence by agreement of KK and POC-CCA trace positive
Village prev. (KK, SAC only)	Village prevalence of infection (KK) among school-age children
Village prev. (POC-CCA, Tr-, SAC only)	Village prevalence of infection (POC-CCA trace negative) among school-age children
Village prev. (POC-CCA, Tr+, SAC only)	Village prevalence of infection (POC-CCA trace positive) among school-age children
Village prev. (KK, Adults only)	Village prevalence of infection (KK) in adults only
Village prev. (POC-CCA, Tr+, Adults only)	Village prevalence of infection (POC-CCA trace positive) among adults
Village prev. (POC-CCA, Tr-, Adults only)	Village prevalence of infection (POC-CCA trace negative) among adults
PHI <5%	Study participant lived in village with prevalence of heavy infection intensity (PHI; 400+ eggs per gram (EPG)) <5% among school-aged children (SAC) and adults
PHI <1%	Study participants lived in village with PHI<1% among SAC and adults
PHI <5% (SAC only)	Study participants lived in village with PHI <5% among SAC only
PHI <1% (SAC only)	Study participants lived in village with PHI <1 % among SAC only
PHI <5% (Adults only)	Study participants lived in village with PHI <5% among adults only
PHI <1% (Adults only)	Study participant lived in village with PHI < 1% among adults only

Table S1 provides all alternative indicators of infection examined in this study. For KK, a single stool sample, double slide thick smears were prepared and read by two technicians with the results from the 41·7mg slides averaged and multiplied by 24 to calculate eggs per gram (EPG). The main outcomes from KK included infection status and intensity. KK infection status was positive when EPG ≥1. WHO infection intensity categories were constructed with none, low, moderate, and heavy as 0, 1-99, 100-399, 400+ EPG respectively. POC-CCA results were interpreted as negative, trace (barely visible test line), positive 1 (test line fainter than control line), positive 2 (test line similar to control line) and positive 3 (test line much darker than control line). Infection status by POC-CCA was positive if positive 1-3 was assigned; two alternative variables were constructed with trace positive (Tr+) and negative (Tr-). Ten percent re-readings of participant diagnoses by a senior technician were completed for KK slides and images of POC-CCA.

Table S2 Descriptive statistics of household and village-level variables for individuals

Variable	**Overall** N = 2834<br>n (%)	**Non-PPF** N = 2492<br>n (%)	**PPF** N = 342<br>n (%)
Home quality score, median (IQR)	6 (3-9)	6 (3-9)	3 (3-7·75)
Household uses safe drinking water source	2281 (80·49)	1990 (79·86)	291 (85·09)
Household has improved sanitation	2031 (71·67)	1788 (71·75)	243 (71·05)
Household has improved hygiene facilities	248 (8·75)	210 (8·43)	38 (11·11)
No. rooms in home, median (IQR)	2 (1 3)	2 (1 3)	2 (1 3)
2+ rooms in home	1972 (69·58)	1734 (69·58)	238 (69·59)
Own a home	2397 (84·58)	2093 (83·99)	304 (88·89)
Home has electricity	1089 (38·43)	964 (38·68)	125 (36·55)
Household purifies drinking water	636 (22·44)	543 (21·79)	93 (27·19)
Household has social status	280 (9·88)	236 (9·47)	44 (12·87)
Household head has social status	236 (8·33)	197 (7·91)	39 (11·40)
Household settlement (yrs), median (IQR)	15 (7 27)	15 (7 26)	20 (9 30)
Village prev. (KK), median (IQR)	41·25 (33·33-56·25)	41·25 (33·33-56·25)	45 (33·75-58·54)
Village prev. (KK)			
11-49	1739 (61·36)	1542 (61·88)	197 (57·60)
50-100	1095 (38·64)	950 (38·12)	145 (42·40)
Village prev. (POC-CCA, Tr-), median (IQR)	40·74 (22·50-53·75)	40·74 (26·25-53·75)	42·50 (20-61·25)
Village prev. (POC-CCA, Tr+), median (IQR)	68·75 (58·75-76·25)	68·75 (58·75-76·25)	66·25 (58·75-73·75)
Village prev. (POC-CCA, Tr-)			
≤30	869 (30·66)	756 (30·34)	113 (33·04)
31-74	1965 (69·34)	1736 (69·66)	229 (66·96)
Village prev. (POC-CCA, Tr+)			
≤30	79 (2·79)	75 (3·01)	4 (1·17)
31-74	1962 (69·23)	1709 (68·58)	253 (73·98)
≥75	793 (27·98)	708 (28·41)	85 (24·85)
Village prevalence (≥ 1 EPG) & (POC-CCA, Tr-), median (IQR)	26·25 (15-37·50)	26·25 (15-37·50)	27·50 (16·25-44·06)
Village prevalence (≥ 1 EPG) & (POC-CCA, Tr+), median (IQR)	35 (26·25-47·50)	35 (26·25-47·50)	36·25 (26·25-50)
PHI <5% (SAC only)	951 (33·56)	859 (34·47)	92 (26·90)
PHI <1% (SAC only)	432 (15·24)	378 (15·17)	54 (15·79)
Village prev. (KK, SAC only)			
≤10	80 (2·82)	67 (2·69)	13 (3·80)
11-49	1539 (54·30)	1378 (55·30)	161 (47·08)
≥50	1215 (42·87)	1047 (42·01)	168 (49·12)
Village prev. (POC-CCA, Tr-, SAC only)			
≤30	748 (26·39)	647 (25·96)	101 (29·53)
31-74	1848 (65·21)	1651 (66·25)	197 (57·60)
≥75-100	238 (8·40)	194 (7·78)	44 (12·87)
Village prev. ≥ 75 (POC-CCA Tr+, SAC only)	1464 (51·66)	1309 (52·53)	155 (45·32)
PHI <5% (Adults only)	753 (26·57)	697 (27·97)	56 (16·37)
PHI <1% (Adults only)	476 (16·80)	433 (17·38)	43 (12·57)
Village prev. (KK, Adults only)	900 (31·76)	784 (31·46)	116 (33·92)
Village prev. ≥ 75 (POC-CCA Tr-, Adult only)	1522 (53·71)	1321 (53·01)	201 (58·77)
Village prev. (POC-CCA, Tr+, Adults only)			
≤30	160 (5·65)	153 (6·14)	7 (2·05)
31-74	2043 (72·09)	1768 (70·95)	275 (80·41)
≥75-100	631 (22·27)	571 (22·91)	60 (17·54)
PHI <5%	1193 (42·10)	1084 (43·50)	109 (31·87)
PHI <1%	80 (2·82)	67 (2·69)	13 (3·80)
Water sites in village			
None	430 (15·17)	366 (14·69)	64 (18·71)
Beach	1029 (36·31)	931 (37·36)	98 (28·65)
Landing site only	1375 (48·52)	1195 (47·95)	180 (52·63)
Public latrine in village	1809 (63·83)	1559 (62·56)	250 (73·10)
District			
Mayuge	959 (33·84)	912 (36·60)	47 (13·74)
Buliisa	950 (33·52)	835 (33·51)	115 (33·63)
Pakwach	925 (32·64)	745 (29·90)	180 (52·63)

*No of households = 1442

A summary of household and village level characteristics is shown for all individuals included in the analysis where the denominator is the number of individuals.

Table S3 Descriptive statistics of villages-level infection indicators

Variable	**N**	**n (%)**
Village prev. (KK), median (IQR)	38	41·25 (33·44-55·94)
Village prev. (KK)	38	
11-49%		24 (63·16)
≥ 50%		14 (36·84)
Village prev. (POC-CCA, Tr-), median (IQR)	38	40·74 (26·88, 53·44)
Village prev. (POC-CCA, Tr+), median (IQR)	38	68·12 (59·06-75·94)
Village prev. ≥31 (POC-CCA, Tr-)	38	
≤ 30%		12 (31·58)
31-74%		26 (68·42)
Village prev. (POC-CCA, Tr+)	38	
≤ 30%		1 (2·63)
31-74%		26 (68·42)
≥ 75-100%		11 (28·95)
Village prevalence (≥ 1 EPG) & (POC-CCA, Tr-), median (IQR)	38	25·62 (16·88- 37·50)
Village prevalence (≥ 1 EPG) & (POC-CCA, Tr+), median (IQR)	38	35·62 (26·56-46·88)
PHI <5% (SAC only)	38	14 (36·84)
PHI <1% (SAC only)	38	7 (18·42)
Village prev. (KK, SAC only)	38	
≤ 10%		1 (2·63)
11-49%		21 (55·26)
≥ 50%		16 (42·11)
Village prev. (POC-CCA, Tr-, SAC only)	38	
≤ 30%		10 (26·32)
31-74%		25 (65·79)
≥ 75%		3 (7·89)
PHI <5% (Adults only)	38	10 (26·32)
PHI <1% (Adults only)	38	6 (15·79)
Village prev. (KK, Adults only)	38	12 (31·58)
Village prev. ≥ 75 (POC-CCA Tr+, SAC only)	38	20 (52·63)
PHI <5%	38	17 (44·74)
PHI <1%	38	1 (2·63)
Water sites in village	38	
None		6 (15·79)
Beach		14 (36·84)
Landing site only		18 (47·37)
Public latrine in village	38	24 (63·16)

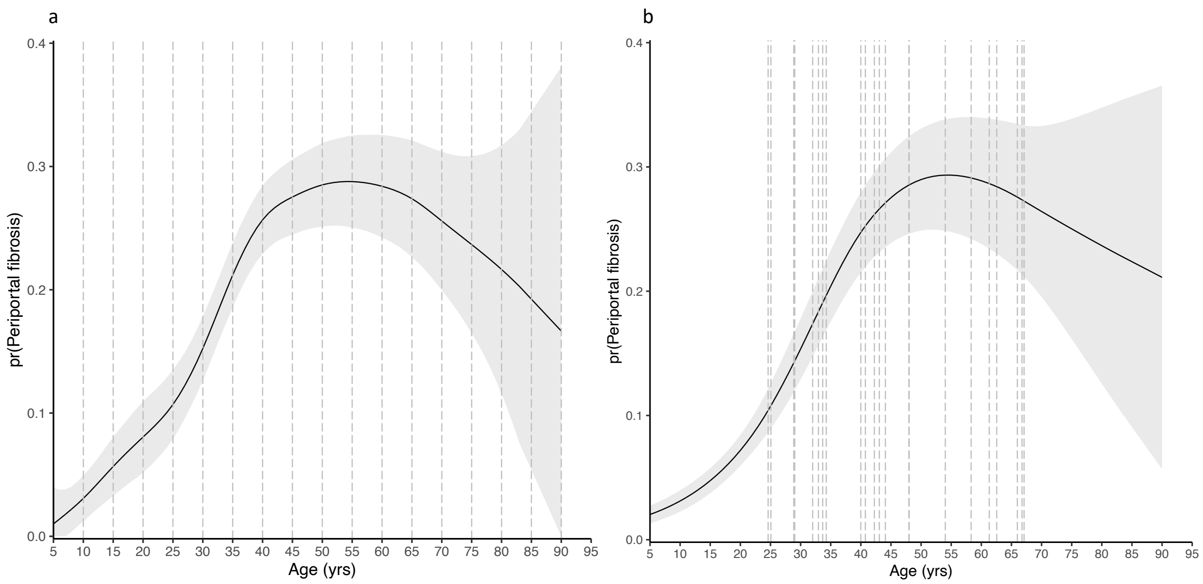

Figure S1 Generalised Additive Model (GAM) with fitted value (logit) of PPF over age. **a**. A GAM is shown with knots placed at every 5 years on the x-axis (age). **b**. A GAM is shown with 22 knots using the "freeknotsplines package in R'. It uses a random search algorithm and generalised cross validation (GCV) for selection of optimised knots numbers and their placement.[8]

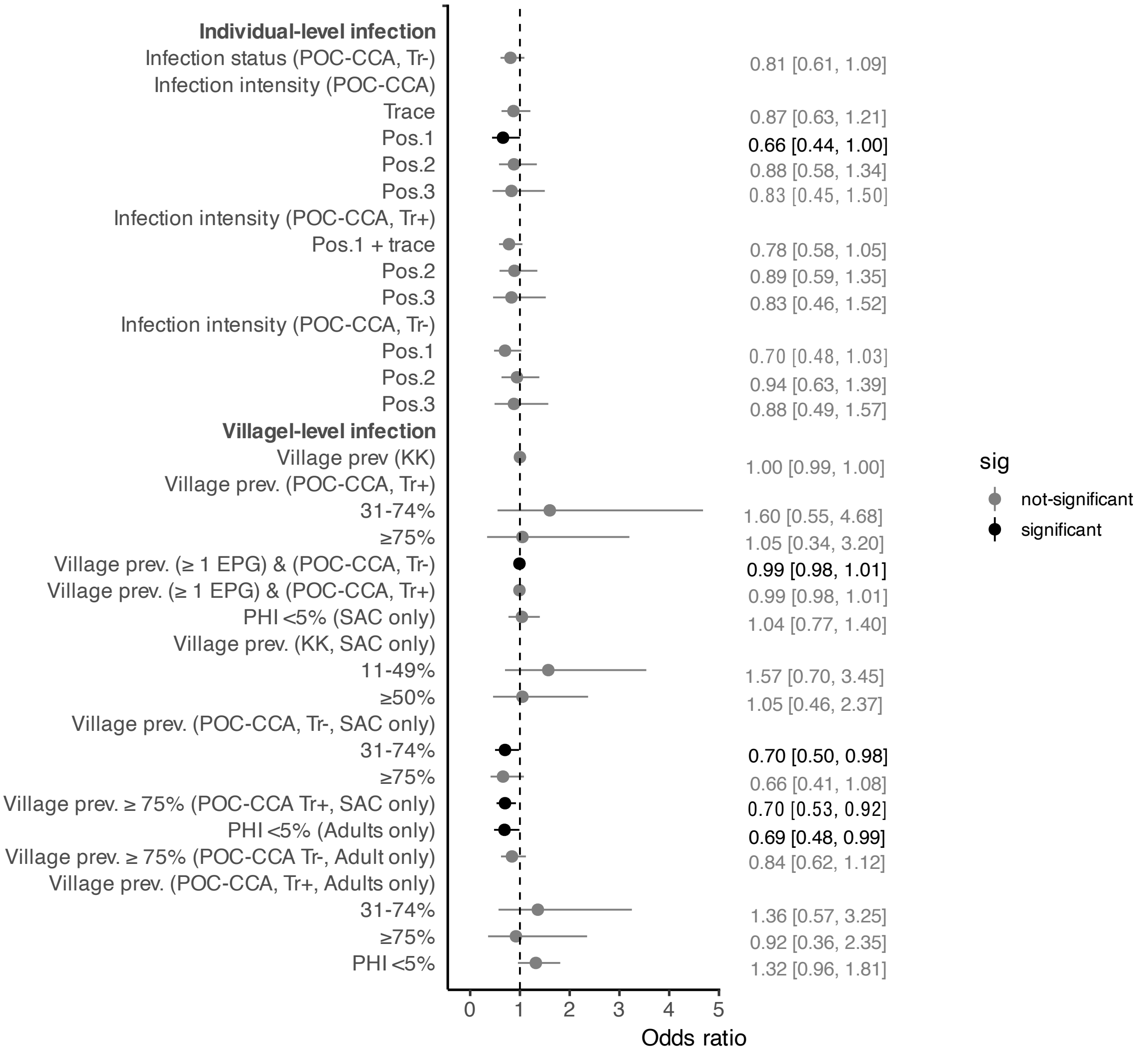

Figure S2 Fully adjusted models with alternative infection indicators. Alternative infection indicators, each representing a separate fully adjusted model, are shown. For each adjusted model, infection status (POC-CCA, Tr+) in Figure 6 was replaced by the other individual-level infection indicators each time while PHI <5% (Adults only) in Figure 6 was replaced by village-level infection indicators at the same time. All models were adjusted for age, gender, religion, tribe, all the medical history indicators, ultrasound-detected chronic hepatitis/early cirrhosis-like disease, malaria diagnosis, water contact, alcohol consumption and smoking status, occupation and all the household level variables in Figure 6. VIFs were between 1·1-2·3. The mean area under the ROC (10-fold CV) in each adjusted model ranged from 0·80-0·83.

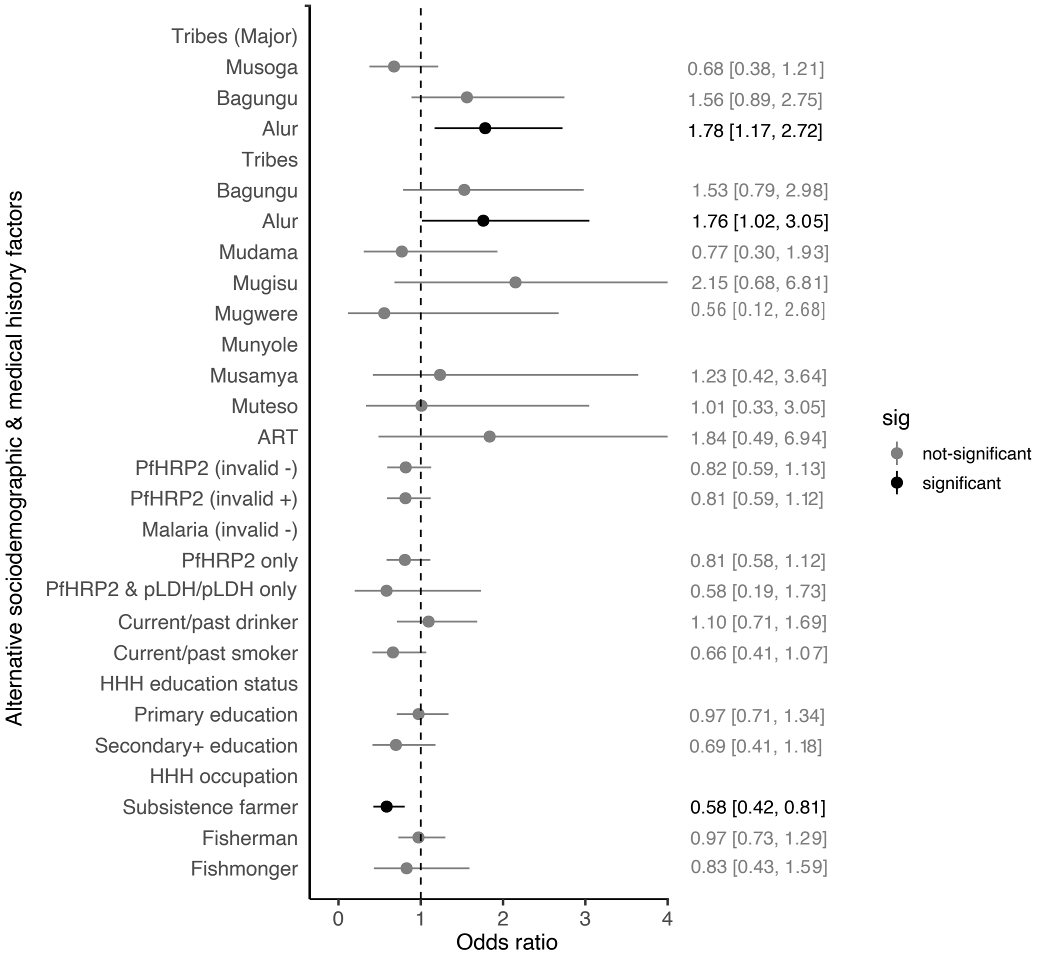

Figure S3 Fully adjusted models with alternative comorbidities and sociodemographic factors. Alternative definitions of sociodemographic and morbidity indicators, especially malaria, are shown where each represent a separate fully adjusted model. Each model was adjusted for age, gender, religion, tribe, all the medical history indicators, ultrasound-detected chronic hepatitis/early cirrhosis-like disease, malaria diagnosis, water contact, current drinker and current smokers, occupation and all the household level variables in Figure 6. VIFs were between 1·1-4·6. The mean area under the ROC (10-fold CV) in each adjusted model ranged from 0·81-0·84. PfHRP2 = Plasmodium falciparum antigen histidine rich protein 2. pLDH = parasite lactase dehydrogenase (generic to all malaria species). For pLDH only, there were only four cases.

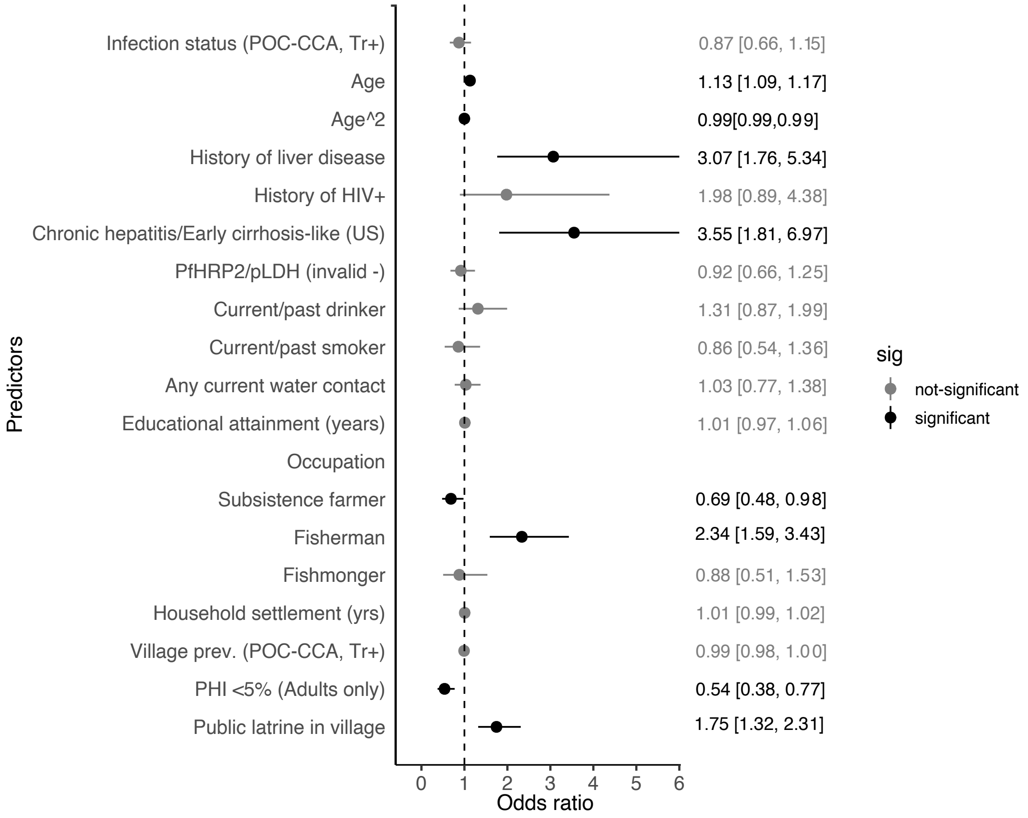

Figure S4 Subgroup analysis of participants from Pakwach District. Obs.= 925. Black represents significant relationships of p-value ≤0·05. VIFs were <10. The mean area under the ROC (10-fold CV) was 0·81. PfHRP2 = Plasmodium falciparum antigen histidine rich protein 2. pLDH = parasite lactase dehydrogenase (generic to all malaria species). For pLDH only, there were only four cases. US = Ultrasound.

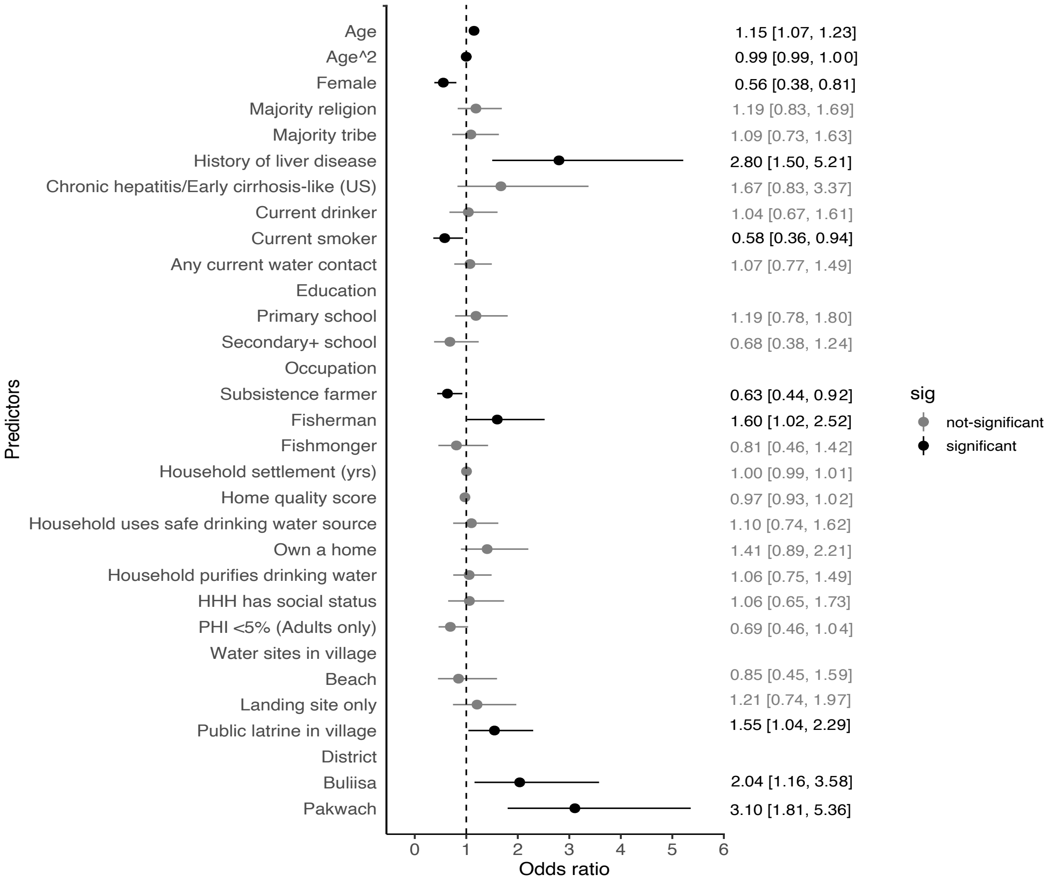

Figure S5 Subgroup analysis of adult participants. Obs. 1426. Black represents significant relationships of p-value≤0·05. VIFs were <10. The mean area under the ROC (10-fold CV) was 0·74. US = Ultrasound

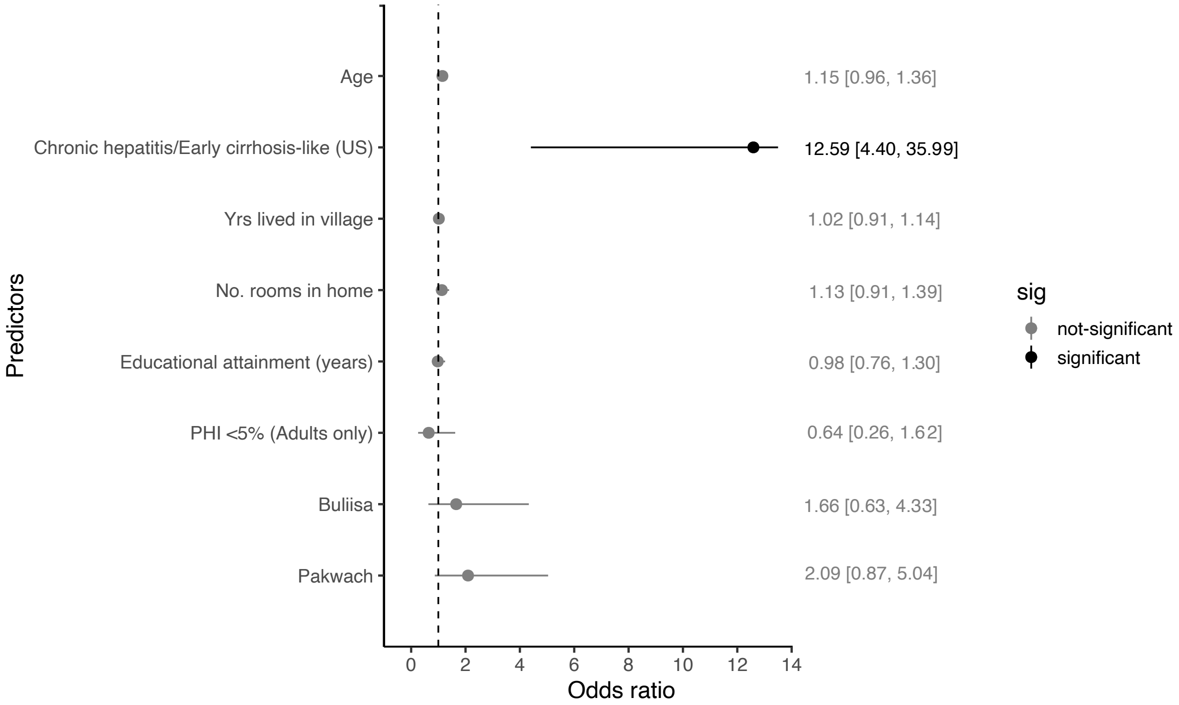

Figure S6 Subgroup analysis of children participants. Obs=1408. Black represents significant relationships of p-value≤0·05. VIFs were l<10. The mean area under the ROC (10-fold CV) was 0·70. US = Ultrasound

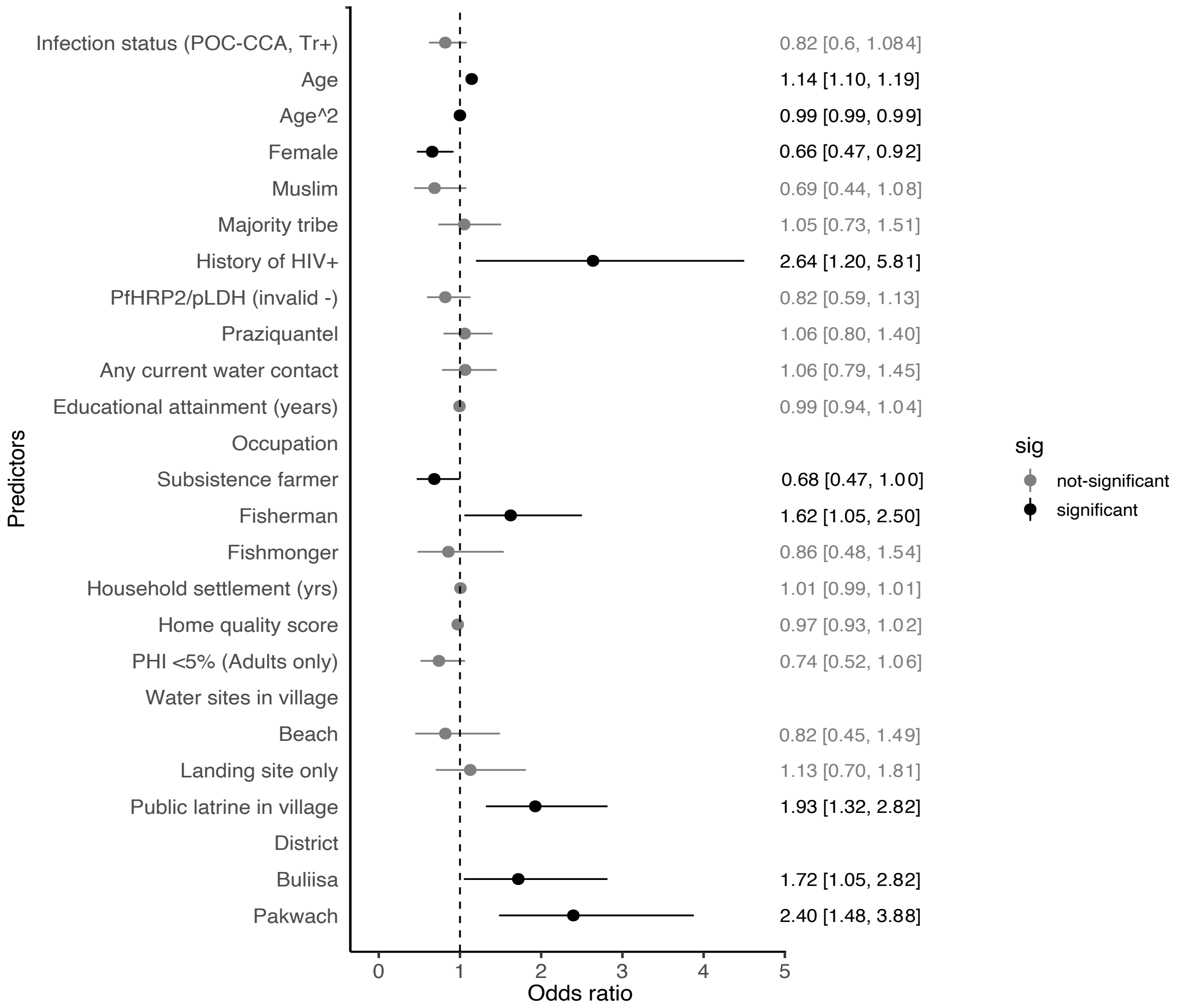

Figure S7 Sensitivity analysis of XY liver patterns. Obs.=2834. Study participants with liver pattern XY (the presence of a comorbidity including ultrasound-detected chronic hepatitis/early cirrhosis-like disease, fatty-like liver or a history of any liver diseases were recoded as zero (no PPF). Black represents significant relationships of p-value≤0·05. VIFs were <10. The mean area under the ROC (10-fold CV) was 0·81. PfHRP2 = *Plasmodium falciparum* antigen histidine rich protein 2. pLDH = parasite lactase dehydrogenase (generic to all malaria species). For pLDH only, there were only four cases.

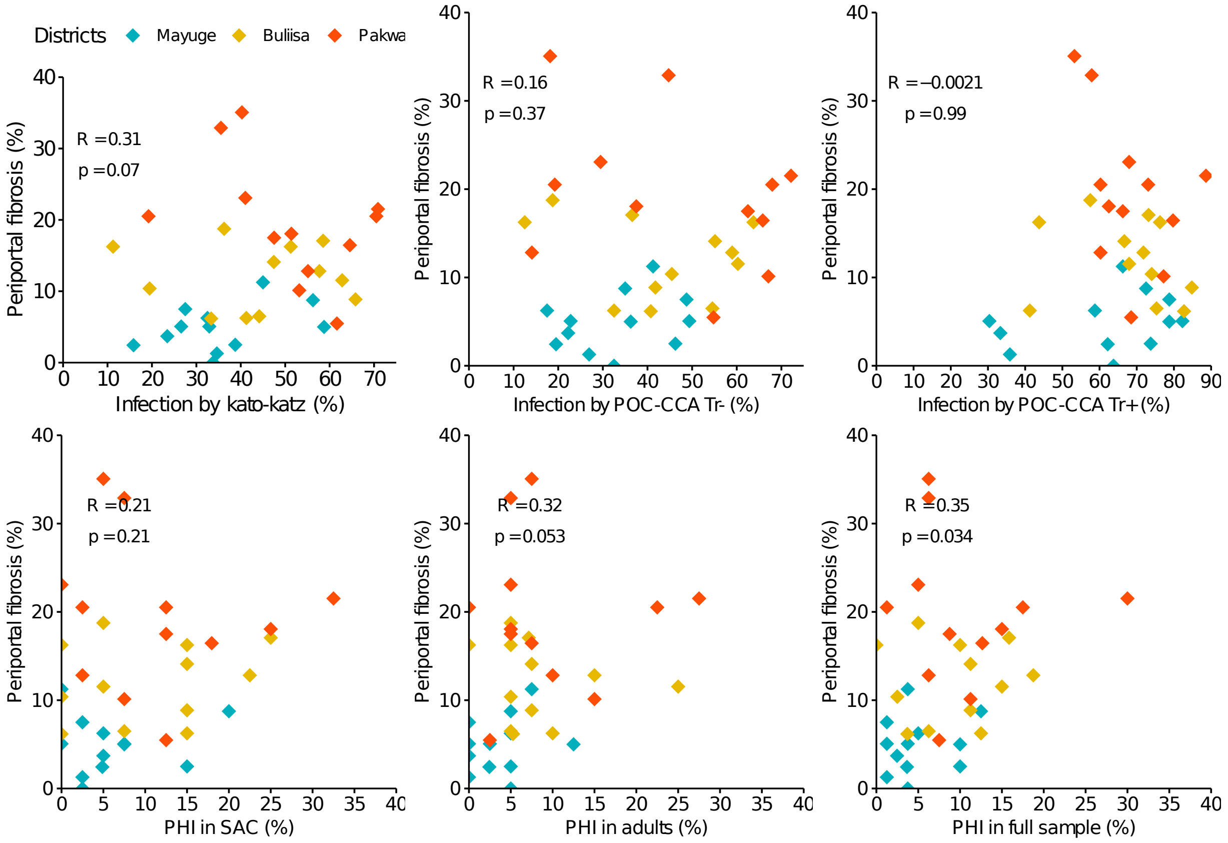

Figure S8 PHI against village PPF prevalence.

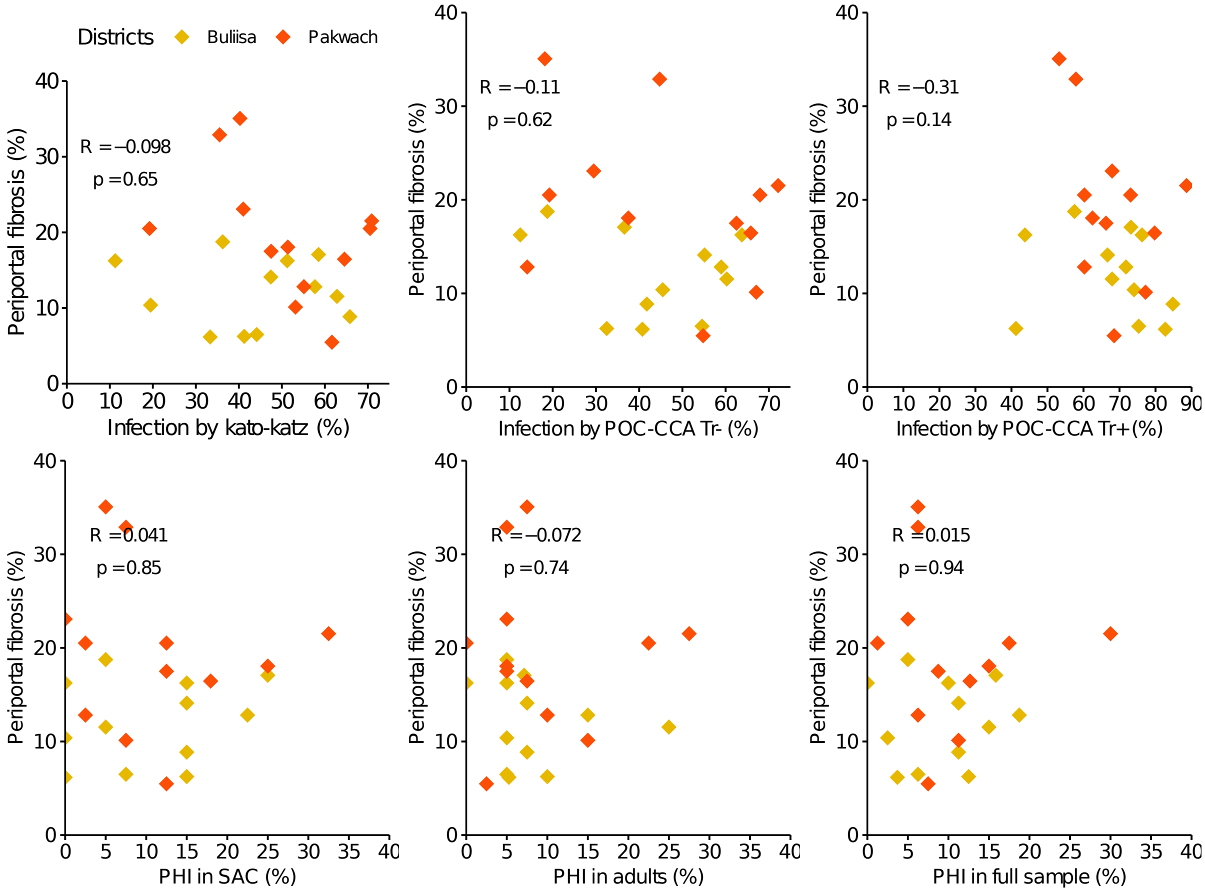

Figure S9 PHI against village PPF prevalence without Mayuge District.

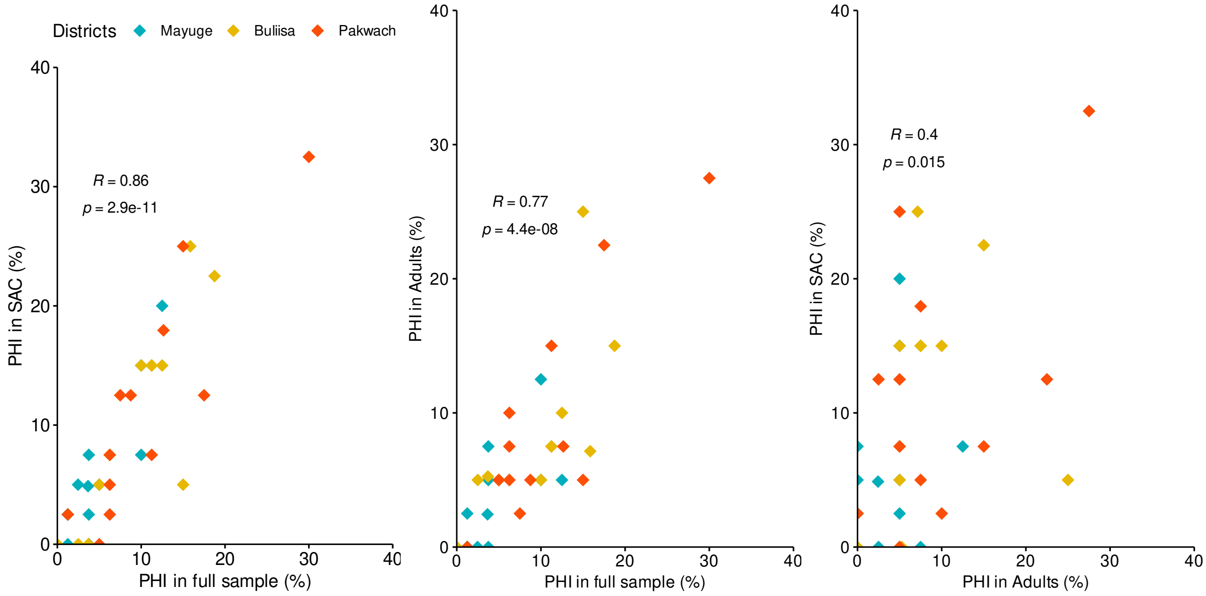

Figure S10 PHI correlations of different age groups.

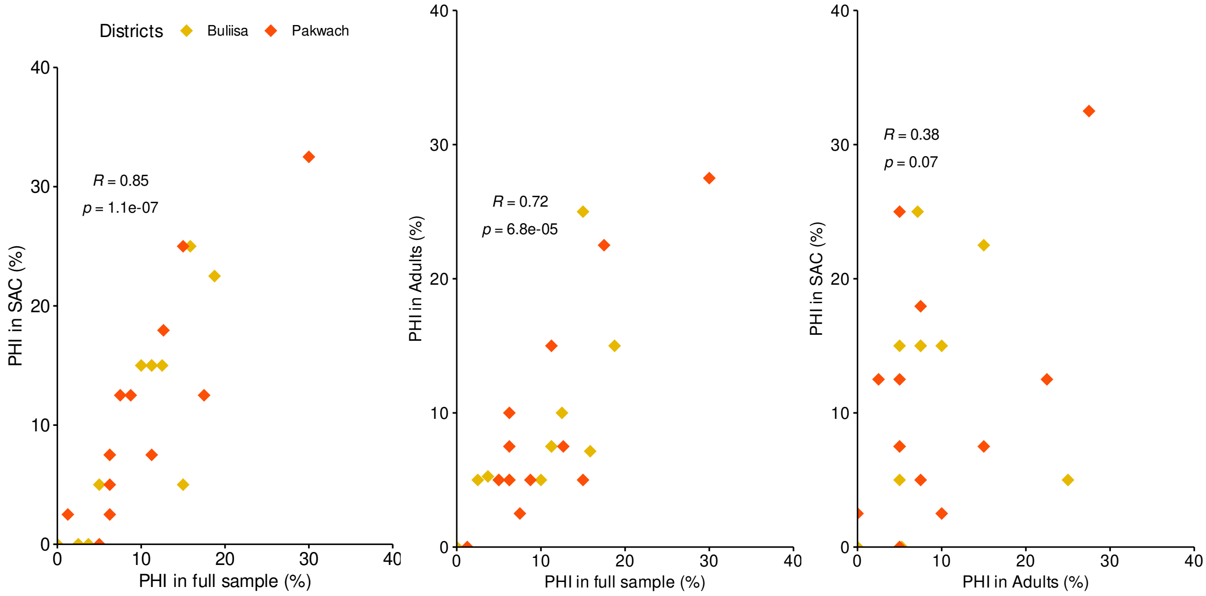

Figure S11 PHI correlations of different age groups without Mayuge District.

References

1. World Health Survey (WHS). WHO Multi-Country Studies Data Archive: an Online Microdata Catalog. 2023. Available at: https://apps.who.int/healthinfo/systems/surveydata/index.php/catalog/whs. Accessed 11 July 2023.

2. World Health Organization. Noncommunicable disease surveillance, monitoring and reporting.: STEPwise approach to NCD risk factor surveillance (STEPS). Available at: https://www.who.int/teams/noncommunicable-diseases/surveillance/systems-tools/steps. Accessed 14 July 2023.

3. Tchelepi H, Ralls PW, Radin R, Grant E. Sonography of diffuse liver disease. *J Ultrasound Med* 2002; **21**:1023–1032.

4. Gaiani S, Gramantieri L, Venturoli N, et al. What is the criterion for differentiating chronic hepatitis from compensated cirrhosis? a prospective study comparing ultrasonography and percutaneous liver biopsy. *J. Hepatol*. 1997; **27**:979–985.

5. Ferraioli G, Monteiro LBS. Ultrasound-based techniques for the diagnosis of liver steatosis. *World J Gastroenterol* 2019; **25**:6053–6062.

6. Chami GF, Kontoleon AA, Bulte E, *et al.* Profiling Nonrecipients of Mass Drug Administration for Schistosomiasis and Hookworm Infections: A Comprehensive Analysis of Praziquantel and Albendazole Coverage in Community-Directed Treatment in Uganda. *Clin Infect Dis* 2016; **62:**200–7.

7. World Health Organization, United Children's Fund. WHO/UNICEF Joint Monitoring Programme for Water Supply, Sanitation, and Hygiene (JMP). 2024. https://washdata.org. Accessed 3 June 2024.

8. Spiriti S, Eubank R, Smith PW, *et al.* Knot selection for least-squares and penalized splines. *JSCS* 2013; **83**:1020–36. doi:10.1080/00949655.2011.647317
